# Platelets cause microvascular occlusion and delayed neurological deficits after subarachnoid hemorrhage in mice

**DOI:** 10.1038/s12276-026-01696-1

**Published:** 2026-04-15

**Authors:** Ari Dienel, Sung-Ha Hong, Kiara Torres, Kanako Matsumura, Jose Guzman, Peeyush Thankamani Pandit, Bibek Samal, Harveen Kaur, Samitha Nemirajaiah, Angelica Bernal, H. Alex Choi, Louise D. McCullough, Spiros L. Blackburn, Jaroslaw Aronowski, Devin W. McBride

**Affiliations:** 1 The Vivian L. Smith Department of Neurosurgery, McGovern Medical School, The University of Texas Health Science Center at Houston, Houston, TX USA; 2 Department of Biosciences, Rice University, Houston, TX USA; 3 Kinesiology Department, Rice University, Houston, TX USA; 4 Cornell University, Ithaca, NY USA; 5 Department of Neurology, McGovern Medical School, The University of Texas Health Science Center at Houston, Houston, TX USA

**Keywords:** Stroke, Cerebrovascular disorders

## Abstract

After subarachnoid hemorrhage (SAH), some patients develop delayed neurological deficits (DND). Microthrombi are considered a contributing factor to DND, but clinical trials of antiplatelets had mixed results. Existing research suggests that platelets play a role in the etiology of DND, but no comprehensive study has tested causality between platelets and DND after SAH. Here we hypothesize that after SAH, platelet activation promotes microthrombi formation, occlusion of the brain microvasculature and contributes to DND, and that inhibiting platelet aggregation is a therapeutic strategy. Mice experiencing SAH were administered various interventions. The animals were subjected to stimulation of platelets, platelet depletion, or antiplatelet drugs and then underwent daily neurological assessment until day 7 before euthanasia for brain microthrombi counting. In parallel, platelets isolated from blood of patients with aneurysmal SAH were tested with antiplatelets to determine how they effected platelet activation. In mice, SAH caused increased levels of platelet-activating factors and more cerebrovascular microthrombi, which correlated with DND. Exogenous platelet activation caused worse outcomes and DND, whereas platelet depletion reduced microthrombi, leading to improved outcomes and a lower incidence of DND. Selected antiplatelet drugs improved outcomes on days 1 and 2 (sex-specific benefits were observed) and one antagonist reduced DND incidence. In platelets from human patients with SAH, antagonism can also prevent platelet activation. After SAH, platelets lead to brain microthrombi and DND, suggesting that platelets are a therapeutic target. Platelet depletion and antagonism can prevent microthrombi and DND in mice. This study serves as a preclinical proof-of-concept that platelets may represent a therapeutic target for SAH.

## Platelets drive microthrombi formation and delayed neurological deficits after subarachnoid hemorrhage, supporting platelet inhibition as a potential therapy

Aneurysmal subarachnoid hemorrhage (SAH) is a severe brain condition with high risks of death and long-term issues. Here researchers explored whether platelets (blood cells that help with clotting) contribute to delayed neurological deficits (DND) after SAH. In this study, they used mice to test whether blocking platelets could prevent DND. They induced SAH in 958 mice and tested various drugs that inhibited platelet function. They found that certain drugs, such as tirofiban, reduced brain microclots and improved outcomes in mice. They also tested human blood samples and found that tirofiban reduced platelet activation. The results suggest that platelets play a deleterious role in DND after SAH and that targeting platelets could be a potential treatment strategy. The study highlights the need for further research to confirm these findings in humans and explore new treatments for patients with SAH. This summary was initially drafted using artificial intelligence, then revised and fact-checked by the author.

## Introduction

Aneurysmal subarachnoid hemorrhage (SAH) is a devastating cerebrovascular disease with high morbidity and mortality^1^. Roughly half of the patients who survive the initial rupture experience delayed neurological deficits (DND) and long-term cognitive disabilities^1^. While the aneurysm rupture accounts for about 25% of the mortality rate, DND, which occurs 4–10 days following SAH, is the most common cause of morbidity and mortality in patients that survive the initial aneurysm rupture^2^. The causes of DND are multifactorial, including microthrombi and vascular occlusion^3–5^. Following aneurysm rupture, it is not surprising that platelets become activated and patients are hypercoagulable. However, patients with SAH who develop DND suffer from persistent platelet activation and hypercoagulability^6–8^. Moreover, clinical studies have reported that patients with SAH who develop DND (compared with patients who do not) have lower platelet counts^9^, are hypercoagulable^10^, have more reactive platelets^11^ and have increased platelet-activating factors, which correlate with the onset of DND^10^, suggesting that platelets may contribute to the pathogenesis of DND. Further supporting this notion, autopsy studies reported that microthrombi are located throughout the brain, localize to regions of infarction and follow a time course mirroring DND^8,12^. The composition of microthrombi in patients with SAH includes aggregated platelets and fibrin^8^. However, whether platelets directly cause DND is not known and has not been extensively studied.

Despite evidence supporting the involvement of platelets in DND pathogenesis, clinical trials have reported mixed results regarding the use of antiplatelet drugs to prevent DND. Early trials on antiplatelet drugs suggested specific drugs may improve outcomes while other antiplatelet drugs had no benefit, but the overall consensus was that antiplatelet therapy may reduce DND and improve outcomes^13,14^. Recent studies and ongoing clinical trials also suggest that antiplatelet drugs may improve outcomes^15,16^, but that some antiplatelet drugs are not suitable^13,14^. It is not clear why some antiplatelet drugs seem to provide benefit while others do not, but it might be due to compensatory activation mechanisms that are stimulated even though one pathway is inhibited^17,18^. Despite the large clinical focus on platelets as a therapeutic target, the mixed findings and unclear treatment targets in humans with SAH suggests that we need a deeper understanding of platelet function and platelet receptors to determine whether platelets are a therapeutic target to prevent DND and improve outcomes after SAH. The current experimental study was designed to test whether platelets are a cause of DND and a therapeutic target for SAH. We hypothesized that platelets cause microthrombi formation, which occlude the brain microvasculature after SAH, thereby inducing DND, and that inhibiting platelet aggregation is a therapeutic target to prevent DND.

## Methods

### Animal study

**Fig. 1:**
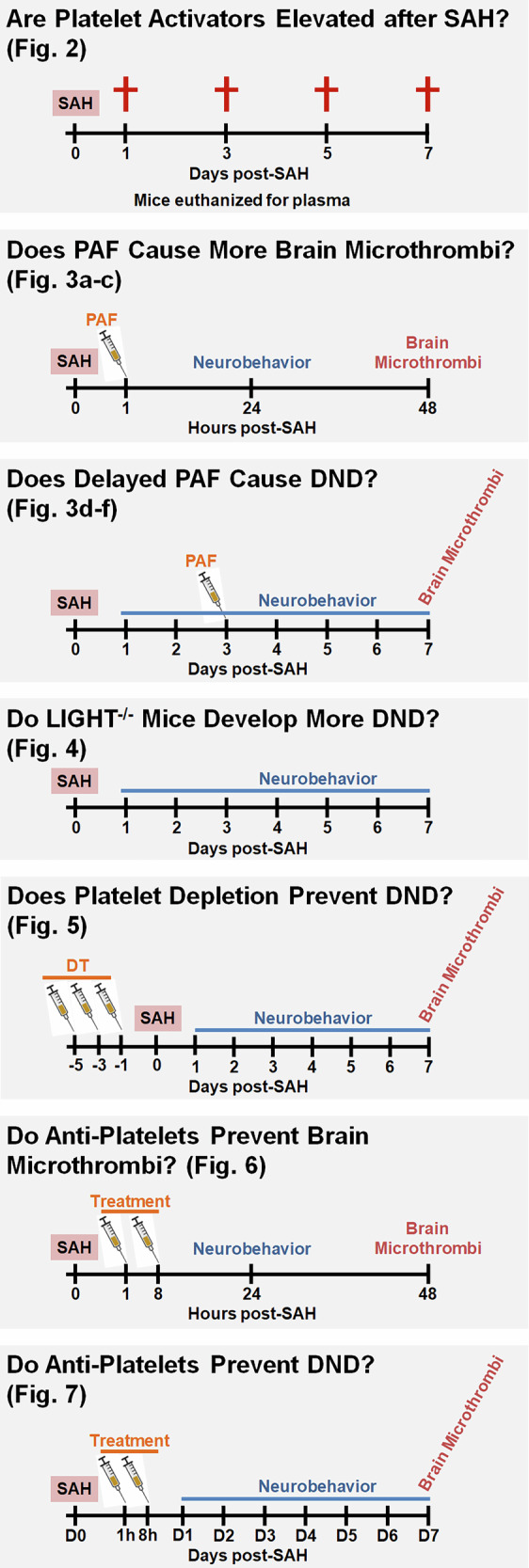
Schematics of the experimental timelines.

A total of 958 mice (4–6 months old) were used. Mice had ad libitum access to food and water and were housed in a temperature-controlled room with a 12-h light–dark cycle. Mice were randomized into groups before use. Confounders were not controlled. Estrous stage was not controlled for in female mice since platelet function does not differ with respect to estrous stage^19^. SPSS was used to estimate all sample sizes using data from previous experiments with *α* = 0.05 and *β* = 0.2. All investigators performing the functional assessment, measurement of outcomes or data analysis were blinded to the sex, genotype and experimental group. A schematic of the experiments is shown in Fig. 1.

SAH, induced using the endovascular perforation model, was confirmed using intracranial pressure monitoring^20,21^ or cessation of breathing (a priori exclusion criteria)^22^. Sham mice underwent all surgical procedures without vessel perforation.

#### PAF induction time course

C57BL6/J (wildtype, WT) mice (*n* = 6 per group per time point) were subjected to either sham or SAH and euthanized on days 1, 3, 5, or 7 post-SAH for collection of blood to measure plasma platelet activators. Blood collected during euthanasia was processed and the plasma was stored at −80 °C. Plasma samples were thawed and used to quantify platelet activating factor (PAF; MBS269434, MyBioSource), thrombin (ab230933, Abcam), thromboxane B_2_ (TXB_2_; 501020, Cayman Chemicals), and platelet factor 4 (PF4; ab100735, Abcam) following the manufacturers’ directions. Samples were tested in triplicate and the mean value was used for graphing statistical analysis.

#### Pharmacologic platelet activation

PAF (4.17 μg/kg in saline; 511075, Calbiochem) was used to activate platelets in WT sham and SAH mice. PAF (or saline) was administered intravenously at 1 h (*n* = 7 sham per sex, *n* = 18 SAH group per sex) or 3 days post-SAH (females only, *n* = 10 sham, *n* = 26 SAH group) for euthanasia on day 2 or 7, respectively, for the quantification of brain microthrombi (*n* = 5–6 per group per sex).

#### Spontaneous platelet activation

LIGHT^−/−^ mice (TNFSF14 knockout) develop increased platelet activation 3 days after wounding and, in this model, platelets are primarily activated by thromboxane A_2_ (ref. ^23^). Therefore, LIGHT^−/−^ mice and WT mice were subjected to SAH (*n* = 25–30 per sex per strain) or sham (*n* = 7 per sex per strain) and allowed to survive for 7 days post-SAH to determine whether LIGHT^−/−^ mice developed DND at a different rate than WT mice.

#### Platelet depletion

PF4-DTR mice (platelets express the diphtheria toxin receptor (DTR)) and WT mice were administered diphtheria toxin (DT, 400 ng intraperitoneally^24^; D0564, Sigma) on days −5, −3 and −1 before SAH to deplete platelets in the PF4-DTR mice. Mice were subjected to sham or SAH and euthanized on either day 2 (*n* = 7 sham per sex per strain, *n* = 10 SAH per sex per strain) or day 7 post-SAH (*n* = 8 sham per sex per strain, *n* = 29–31 SAH per sex per strain). Brains were used for quantification of microthrombi (*n* = 5–6 per sex per group). Blood was collected during euthanasia to measure the platelet count and to confirm platelet depletion in PF4-DTR mice.

#### Platelet inhibition

WT mice were subjected to either sham or SAH and then euthanized at 2 (*n* = 10 per sex per group) or 7 days post-SAH (females only, *n* = 10 sham, *n* = 20/SAH group). Platelet antagonists or vehicles were administered intravenously at 1 and 8 h post-SAH. The inhibitors used were A3P5PS (P2Y_1_ antagonist, 1 mM (ref. ^25^); A1651, Sigma) combined with clopidogrel (P2Y_12_ antagonist, 20 mg/kg (ref. ^25^); S1415, Selleckchem), daltroban (thromboxane receptor antagonist, 5 mg/kg (ref. ^26^); D7441, Sigma), ML354 (PAR-4 antagonist, 10 mg/kg (ref. ^27^); 5387, Tocris), WEB2086 (PAF receptor antagonist, 10 mg/kg (ref. ^28^); 2339, Tocris), and tirofiban (GPIIb/IIIa antagonist, 0.25 mg/kg (ref. ^29^); S8594, Selleckchem). Ethanol (25% in saline) was used as the vehicle for A3P5PS + clopidogrel, daltroban, ML354, and WEB2086. Saline was used as the vehicle for tirofiban. Brains were used for quantification of microthrombi (*n* = 6 per sex per group).

#### Neurobehavior and DND

Mice were subjected to daily neurobehavioral assessment using a well-validated composite neuroscore scale (that tests sensory and motor ability)^30^. Mice that had a neuroscore of 8 or less on day 1 were excluded and replaced (a priori exclusion criteria). The neuroscores from individual mice were used to determine whether the mouse developed DND. DND was defined as follows: (1) mice must have some recovery from the day 1 neuroscore and (2) after some recovery, mice had to experience a worsening neuroscore that is 5 or more points less than their best performance (from any prior post-SAH day)^31^. Animals experiencing delayed death (after some functional recovery) were also considered as developing DND since their neuroscore would be equal to 0. Supplementary Table 1 presents representative data from mice developing DND and those not developing DND.

#### Histological staining for microthrombi

At the time of euthanasia, mice were deeply anesthetized and then perfused with PBS via cardiac puncture followed by 4% PFA perfusion. The brains were removed and stored until being sectioned into 40-μm-thick slices with a vibratome. Slices at −2 from bregma were stained with Martius, Scarlet and Blue (MSB) to visualize microthrombi using a FLEXACAM (color camera) on a DMi8 with a 20× objective and individual images were stitched together using the LASX software. Microthrombi, counted for the entire slice, were identified as red line segments and each vessel branch containing a red line segment was counted as a single microthrombi^31^ (Supplementary Fig. 1).

#### Statistical analysis

Data are presented as individual data points with the mean and standard deviation (unless otherwise stated). All data were assessed for normality and homoscedasticity and *P* < 0.05 was considered statistically significant. Individuals performing analysis were blinded to sex, genotype, and experimental group. One-way or two-way analyses of variance (with repeated measures as appropriate) or two-tailed unpaired *t*-tests were performed. Data were corrected for multiple comparisons using the appropriate post hoc tests. DND incidence (see the Supplementary Information for the equation) was analyzed by a log-rank test. Correlational analysis for microthrombi counts, clusters, neuroscore, and DND were performed using Spearman and Pearson correlational coefficients as appropriate. Graphpad Prism and SPSS 28 were used for graphing and analysis. Mortality and excluded animals are reported in the Supplementary Information. Statistical reports for all outcomes can be found in Supplementary Tables 9–26.

### Human study

Sixteen patients with aneurysmal SAH were enrolled into a prospective observational study approved by the Institutional Review Board at UTHealth. Blood samples were collected into sterile BD vacutainers ACD-B (364816, Becton Dickinson) on days 1, 2, 4, 7, and 10 after SAH, deidentified and then given to the laboratory. The patient demographics are reported in Supplementary Table 7.

#### Platelet morphology

Blood was processed within 30 min after it was drawn, as previously described^32^. Briefly, the blood was mixed with wash buffer (1:1 ratio, the buffer contained 10 mM sodium citrate, 150 mM NaCl, 1 mM EDTA and 1% (w/v) dextrose in Tyrode’s buffer (11760-10, EMS) at pH 7.4) and then underwent a series of centrifugations. The supernatant was then carefully aspirated until about 100 μl remained with the pellet, 1500 μl of platelet washing buffer was added and the tube was flicked three times to resuspend the pellet. The tube was centrifuged for 8 min at 100*g* and room temperature and then the supernatant was collected and the platelets counted (Hemavet 950 FS, Drew Scientific). Following platelet counting, 750 μl of the platelet sample was put into two tubes. One tube had 100 μl of either A3P5PS (1.5 mM)^25^ or tirofiban (50 μM) added and the other tube had 100 μl of saline added, the tubes were flicked three times and then incubated for 15 min at 37 °C. Each sample was placed onto poly-L-lysin-coated glass slides and incubated for 30 min at 37 °C. Slides were washed with PBS twice, fixed with 4% PFA for 15 min and then 0.3% Triton-X100 was added for 10 min followed by incubation with Phalloidin-647 (1:1,000, ab176759, Abcam) for 2 h. The samples were then washed and dried in the dark overnight at room temperature. The samples were carefully placed onto microscope slides using Fluoromount-G. Fluorescence images were taken over the whole area at 100×. Platelet morphology was categorized as either not activated or activated^32–34^.

#### Statistical analysis

The individuals performing the statistical analysis were blinded to experimental condition, time point, and patient demographics. Univariate analysis was used for testing the differences of the variable distributions between the experimental conditions.

### Study approval

Animal use was approved by the UTHealth Animal Welfare Committee and conducted in compliance with the NIH Guidelines for the Use of Animals in Neuroscience Research. Data are reported in compliance with the ARRIVE guidelines. The human study was approved by the UTHealth Institutional Review Board.

## Results

### Plasma levels of Platelet-Activating Factors are elevated after SAH

**Fig. 2:**
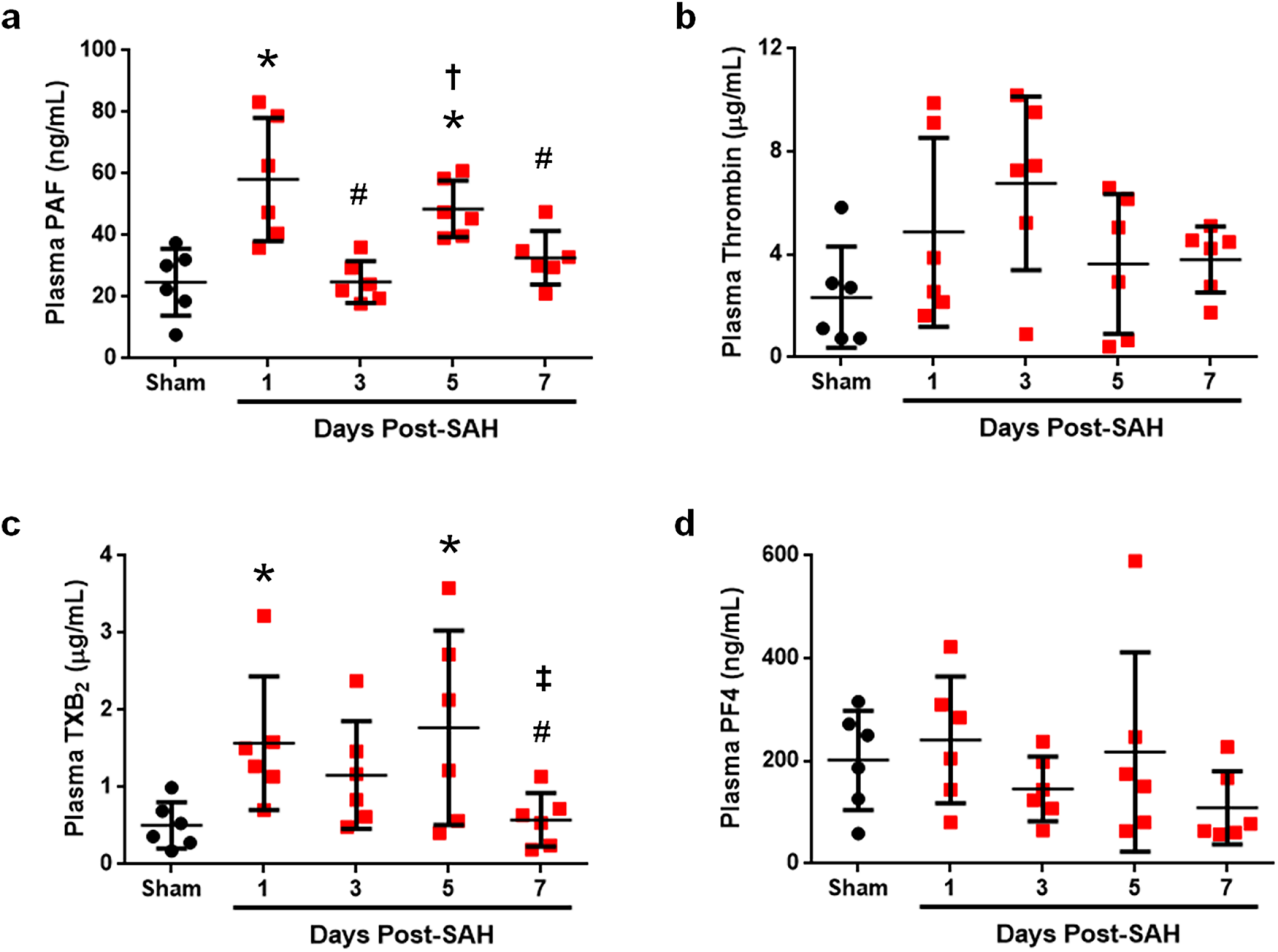
Plasma PAF and TXB_2_ are elevated on days 1 and 5 post-SAH. **a**–**d** Levels of plasma PAF (**a**), thrombin (**b**), TXB_2_ (**c**), and PF4 (**d**). Female mice were used (*n* = 6 per group per time point). *P < 0.05 versus sham, ^#^*P* < 0.05 versus SAH day 1, ^†^*P* < 0.05 versus SAH day 3, and ^‡^*P* < 0.05 versus SAH day 5.

We began testing our overall hypothesis on the underlying role of platelet activation as a cause of DND by measuring the plasma levels of four key factors regulating the activation of platelets (PAF, thrombin, TXB_2_, and PF4). We established that SAH in mice caused statistically significant increases in PAF and TXB_2_ on days 1 and 5. Although a trend was observed, neither thrombin nor PF4 were significantly elevated after SAH within 5 days post-SAH (Fig. 2). These results suggest potential dysregulation of platelet activation after SAH.

### Exogenous platelet activation worsens outcome

**Fig. 3:**
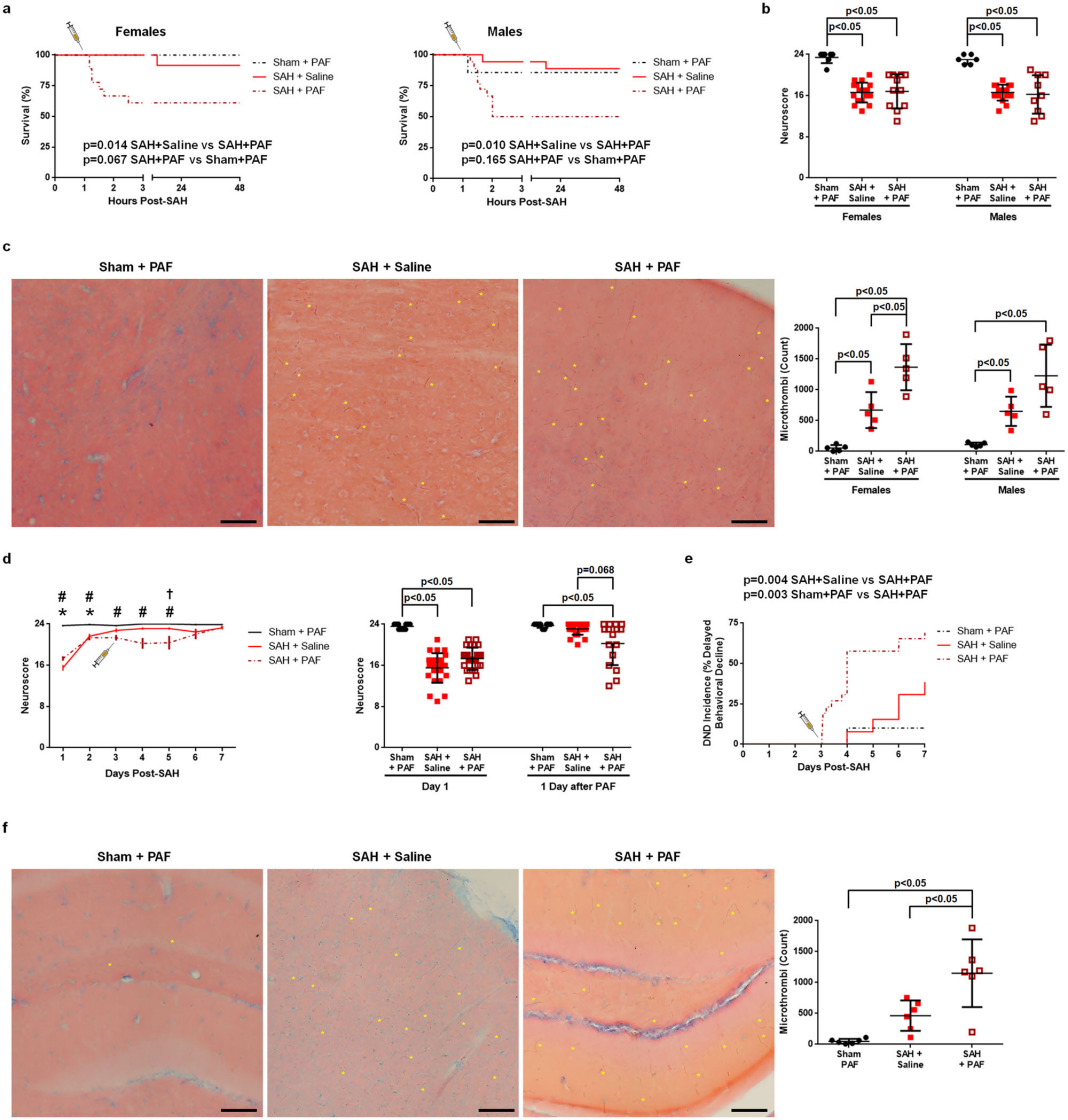
Exogenous PAF worsens outcomes after SAH in mice. **a–c** PAF administered at 1-h post-SAH causes higher mortality (**a** Sham + PAF *n* = 7 per sex, SAH + saline *n* = 18 per sex, SAH + PAF *n* = 18 per sex) and more brain microthrombi (**c** *n* = 5 per sex per group) than saline-treated female and male mice. Neuroscore (**b**) was similar for SAH + saline (females: *n* = 18, males: *n* = 18) and SAH + PAF (females: *n* = 11, males: *n* = 9), and both SAH groups had neuroscores significantly lower than Sham+PAF (females, *n* = 7; males, *n* = 6). **d**–**f** In female mice, PAF administered 3 days after SAH causes worse neurological behavior (**d** mean with s.e.m., sham + PAF *n* = 9–10, SAH + saline *n* = 16–26, and SAH + PAF *n* = 13–26), a higher rate of DND (**e** sham + PAF *n* = 10 and SAH *n* = 26) and more brain microthrombi (**f** *n* = 6 per group). Representative images of MSB-stained brain slices (**c** and **f** scale bar=100 μm) showing microthrombi (selected microthrombi marked with yellow asterisks) are best observed when zoomed in. *P < 0.05 sham + PAF versus SAH + saline, ^#^*P* < 0.05 sham + PAF versus SAH + PAF, and ^†^*P* < 0.05 SAH + saline versus SAH + PAF.

Since PAF plays an important role in platelet activation and the levels of PAF are increased after SAH, we tested the hypothesis that administering exogenous PAF worsens outcome and triggers DND. In the first experiment, we injected PAF at 1-h post-SAH using a dose that elevates the plasma PAF by 70 ng/ml, which is comparable to levels on days 1 and 5 post-SAH in mice. Compared with saline, PAF led to early (within 2 h) death and a significantly higher mortality rate in both female and male SAH mice (18/36 SAH + PAF versus 3/36 SAH + saline) (Fig. 3a and Supplementary Table 3). Mice that survived the response to PAF did not have a significantly worse neuroscore compared with saline-treated mice on day 1 after SAH (Fig. 3b), but PAF increased brain microthrombi as measured on day 2 (Fig. 3c). These findings reveal an acute deleterious effect of PAF, but whether PAF drives delayed neurological deficits remains unclear.

Because PAF injection 1 h after SAH produced high mortality that prohibited the accurate assessment of neurological outcome, we then performed a 7-day study to determine whether delayed PAF (administered on day 3 post-SAH) would worsen outcomes and increase DND incidence. Indeed, mice treated with PAF on day 3 had a significantly lower neuroscore on day 4 (24 h after PAF injection) and a significantly higher incidence of DND compared with saline-treated SAH mice, which corresponded with more brain microthrombi (Fig. 3d–f).

**Fig. 4:**
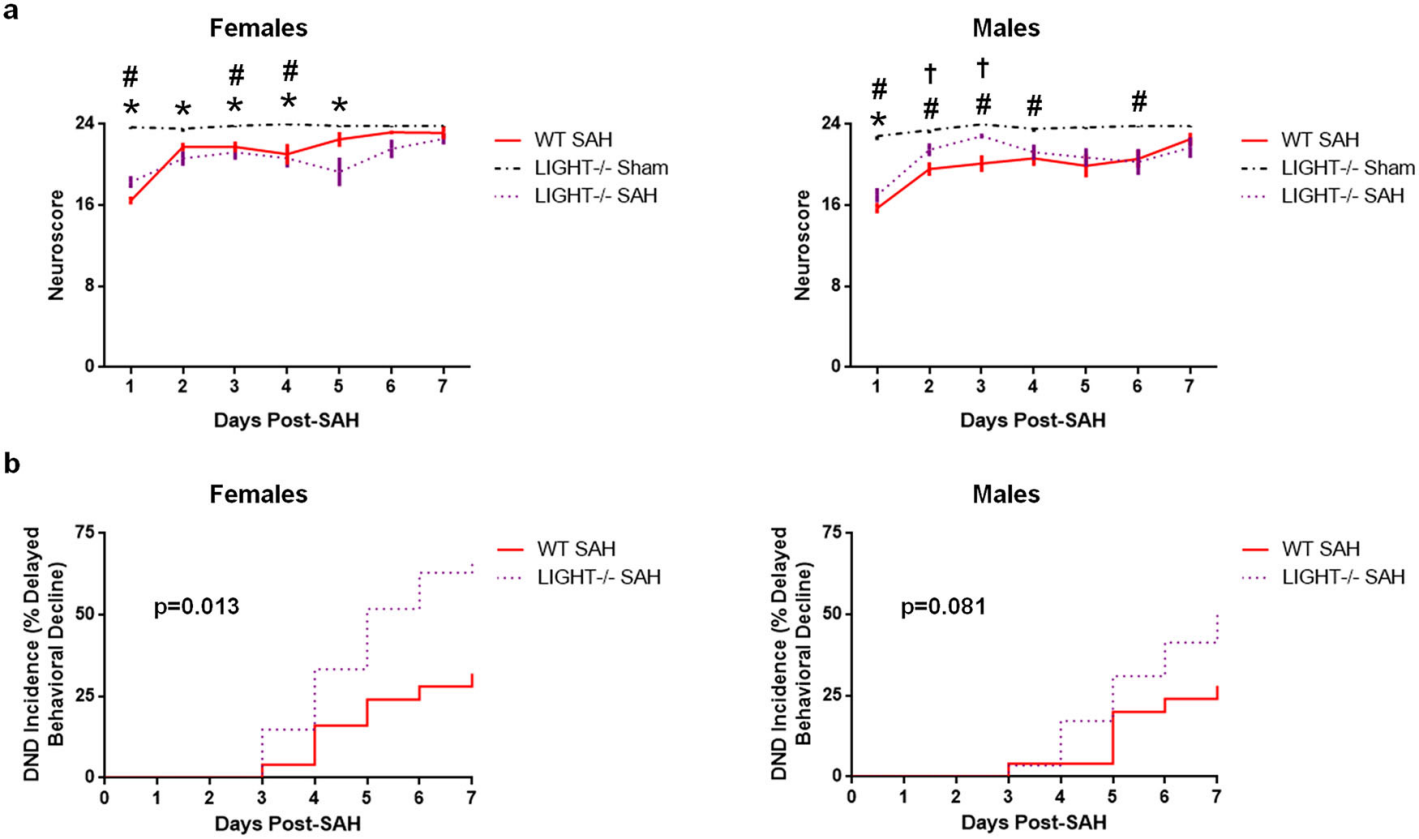
Delayed platelet activation causes DND after SAH. **a**, **b** LIGHT^−/−^ mice, which develop platelet activation 3 days after injury, have a higher DND incidence than WT mice. For the neuroscore (**a** mean with s.e.m.), sham *n* = 7 per sex, female WT SAH *n* = 19–25, female LIGHT^−/−^ SAH *n* = 14–30, male WT SAH *n* = 19–25, and male LIGHT^−/−^ SAH *n* = 21–29. For the incidence of DND (**b**), WT SAH *n* = 25 per sex, female LIGHT^−/−^ SAH *n* = 27, and male LIGHT^−/−^ SAH *n* = 29. *P < 0.05 LIGHT^−/−^ sham versus LIGHT^−/−^ SAH, ^#^*P* < 0.05 WT sham versus WT SAH, and ^†^*P* < 0.05 LIGHT^−/−^ SAH versus WT SAH.

To further implicate the role of platelets in DND, we next utilized LIGHT^−/−^ mice, which are known to develop activated platelets 3 days after being subjected to injury^23^ and which is probably caused by increased arachidonic acid metabolites^35^. Following SAH, despite having day 1 neuroscores that were indistinguishable from WT SAH mice, SAH LIGHT^−/−^ mice developed a significantly higher incidence of DND (Fig. 4), suggesting that delayed platelet activation may contribute to DND.

### Platelet depletion improves outcomes and reduces DND

**Fig. 5:**
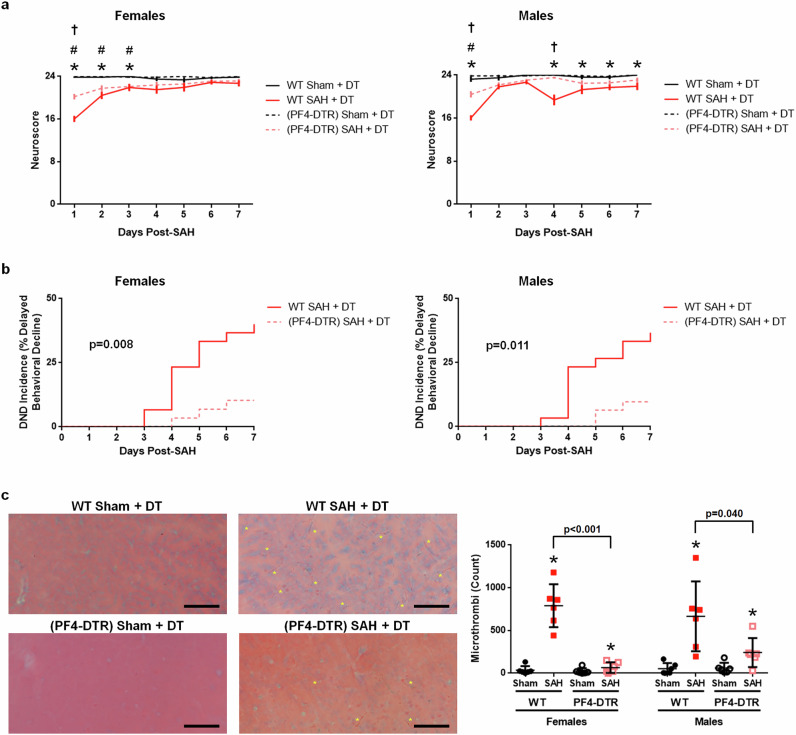
Platelet depletion reduces DND after SAH. **a**–**c**, After SAH, mice depleted of platelets (PF4-DTR + DT), compared with nondepleted (WT + DT) mice, had better neuroscore (**a** mean with s.e.m.), less DND (**b**), and fewer brain microthrombi (**c**) (scale bar = 100 μm, representative images of MSB-stained brain slices with yellow asterisks marking selected microthrombi (best observed zoomed in)). All mice received the entire DT regimen. For neuroscore (**a**), sham *n* = 8 per sex, female WT SAH *n* = 20–30, female (PF4-DTR) SAH *n* = 27–29, male WT SAH *n* = 25–30, and male (PF4-DTR) SAH *n* = 30–31. For DND (**b**), WT SAH *n* = 30 per sex, and (PF4-DTR) SAH *n* = 29 female and *n* = 31 male. *P < 0.05 WT sham versus WT SAH, ^#^*P* < 0.05 (PF4-DTR) sham versus (PF4-DTR) SAH, and ^†^*P* < 0.05 WT SAH versus (PF4**-**DTR) SAH.

As brain microthrombi are increased after SAH and may cause DND, and since platelet activation promotes DND (as shown above), we next tested the hypothesis that platelets are a key cell type contributing to DND after SAH. Thus, with transgenic mice expressing the DTR on platelets (PF4-DTR mice), we depleted platelets by injecting DT three times in naive mice but DT did not deplete platelets in WT mice (Supplementary Fig. 5). In our experiment, both WT and PF4-DTR mice received DT before SAH and we confirmed that only the PF4-DTR mice showed platelet depletion (Supplementary Fig. 6). In agreement with the detrimental role of platelets, mice depleted of platelets showed a significantly improved neuroscore at 1 day post-SAH. This neurological improvement corresponded with less brain microthrombi on day 2 (Supplementary Fig. 18). Importantly, in male and female SAH mice, platelet depletion was associated with a significantly reduced incidence of DND and with fewer brain microthrombi on day 7 (Fig. 5). Taken together, platelet depletion not only improved acute neuroscore deficits, but also reduced DND incidence, further implicating platelets as a cause of DND after SAH.

### Platelet antagonism can improve SAH outcome and reduce DND

**Fig. 6:**
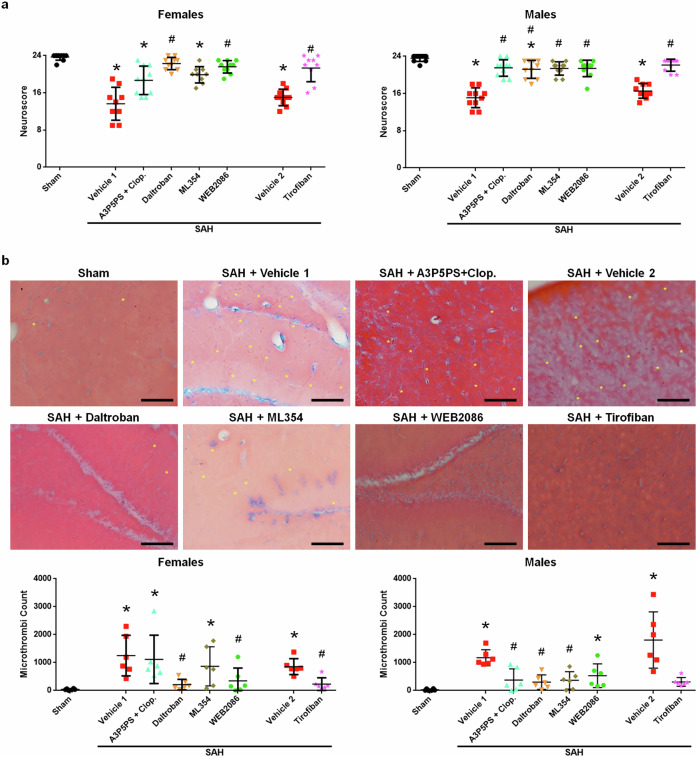
Platelet antagonists can improve outcomes after SAH. Mice were euthanized 2 days after SAH. **a** Day 1 neuroscore (*n* = 9–10 per sex per group). **b** Day 2 brain microthrombi count (*n* = 6 per sex per group) (scale bar = 100 μm, representative images of MSB-stained brain slices with yellow asterisks marking selected microthrombi are best observed zoomed in). *P < 0.05 versus sham and #*P* < 0.05 versus SAH + vehicle 1 or 2, as appropriate.

While platelet depletion in mice after SAH improved the outcomes and reduced the incidence of DND, platelet depletion in humans with SAH may not be clinically practical. Thus, to determine the clinical relevance, we explored whether platelet inhibitors could provide early clinical benefit and also prevent microthrombi and DND after SAH. For this, we compared the efficacies in reducing SAH-induced injury and microthrombi formation of five drugs that block different receptors known to cause platelet activation (antagonism of purinergic G protein-coupled P2Y receptors by A3P5PS and clopidogrel, thromboxane A_2_ receptor inhibition by daltroban, PAR-4 antagonism by ML354, and PAF receptor inhibition by WEB2086) and an inhibitor (tirofiban) for a platelet aggregation receptor (glycoprotein IIb/IIIa (GPIIb/IIIa)). When administered at 1 and 8 h after SAH, all five interventions significantly improved the day 1 neuroscore in male SAH, and all but WEB2086 significantly reduced brain microthrombi (Fig. 6a). Interestingly, in female SAH mice, only inhibition of thromboxane A_2_ receptor (daltroban), PAF receptor (WEB2086), and GPIIb/IIIa (tirofiban) significantly improved the day 1 neuroscore, which corresponded with reduce microthrombi counts (Fig. 6b). However, female SAH mice did not benefit from inhibition of P2Y receptors (A3P5PS and clopidogrel) or PAR-4 (ML354).

**Fig. 7:**
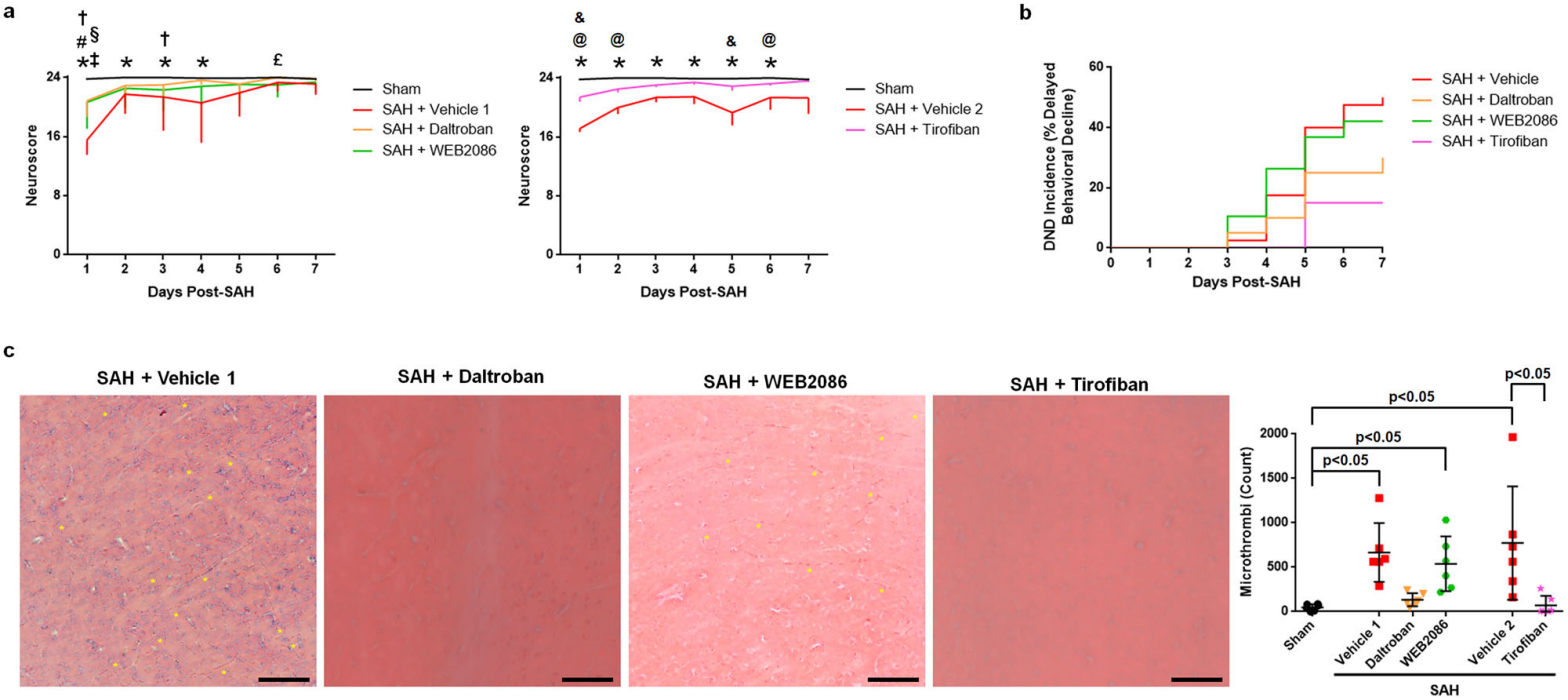
Tirofiban reduces microthrombi and DND after SAH. Female mice were allowed to survive for up to 7 days post-SAH. **a** For neuroscore, sham *n* = 10, SAH + vehicle 1 *n* = 10–20, SAH + vehicle 2 *n* = 15–20, SAH + daltroban *n* = 15–20, SAH + WEB2086 *n* = 14–20, and SAH + tirofiban *n* = 18–20. *P < 0.05 sham versus SAH + vehicle (1 or 2 as appropriate), ^#^*P* < 0.05 sham versus SAH + daltroban, ^†^*P* < 0.05 sham versus WEB2086, ^‡^*P* < 0.05 SAH + vehicle 1 versus SAH + daltroban, ^§^SAH + vehicle 1 versus SAH + WEB2086, ^£^SAH + daltroban versus SAH + WEB2086, ^@^*P* < 0.05 sham versus SAH + tirofiban, and ^&^*P* < 0.05 SAH + vehicle 2 versus SAH + tirofiban. **b** For DND, SAH + vehicle *n* = 40, and *n* = 20 for each antiplatelet group. **c** Day 7 brain microthrombi count (*n* = 6 per group; scale bar=100 μm, representative images of MSB-stained brain slices with yellow asterisks marking selected microthrombi are best observed zoomed in).

Since only daltroban, WEB2086, and tirofiban benefited both sexes, to identify a therapeutic that could work in males and females, we then tested whether any of these three platelet antagonists could reduce DND. In agreement with the 2-day study, all three platelet antagonists improved neuroscore on day 1 after SAH in female mice (Fig. 7a). Although daltroban showed a strong trend toward DND reduction (*P* < 0.159 compared with vehicle) with reduced brain microthrombi formation, WEB2086 did not lower DND incidence or microthrombi. However, only the platelet aggregation antagonist, tirofiban, significantly reduced DND (*P* = 0.009 compared with vehicle-treated mice) as well as the number of brain microthrombi on day 7 in female SAH mice (Fig. 7).

### Inhibiting human platelets

**Fig. 8:**
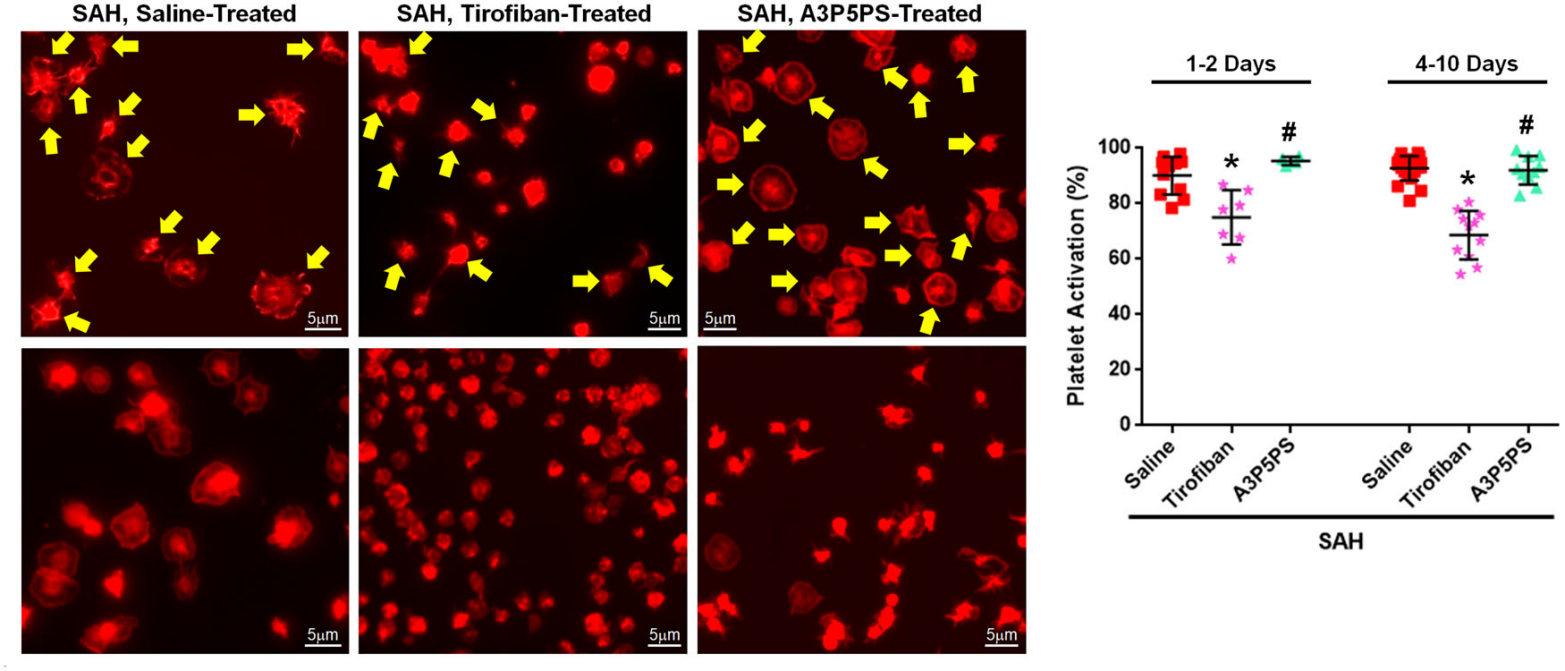
Tirofiban reduces platelet activation in human aneurysmal SAH samples. Representative images show the platelet morphology changes (activated platelets). Selected activated platelets are marked with a yellow arrow. *P < 0.05 versus SAH + saline and ^#^*P* < 0.05 versus SAH + tirofiban.

Now that tirofiban was identified as a potential treatment for DND in SAH mice, we next examined tirofiban for any effect on human platelets. We collected blood from patients with aneurysmal SAH, isolated the platelets, cultured them with a platelet antagonist or saline, and quantified the platelet activation via morphological analysis (that is, spreading). Saline-treated platelets were primarily activated (as shown by platelet spreading at 1–2 days post-SAH, 90.2%; 4–10 days post-SAH, 92.8%) and the P2Y_1_ antagonist A3P5PS did not attenuate platelet spreading. However, tirofiban significantly reduced platelet spreading in patient samples collected early (1–2 days) and during the DND period (4–10 days) (Fig. 8). Taken together with the results from our mouse study, GPIIb/IIIa inhibition is a promising therapeutic target.

## Discussion

Although there have been prior preclinical studies proposing individual platelet inhibitors as a treatment for SAH^36–38^, there has not been a comprehensive and comparative study of the role of platelets in DND, nor has there been any direct evidence that platelets cause DND. Herein, we systematically tested the hypothesis that, after SAH, platelets cause DND via microthrombi-mediated occlusion of brain microvessels and that approaches aimed at inhibiting platelets are a therapeutic target to prevent DND. Here, using a clinically relevant experimental model of SAH, we first showed an early (day 1) and a late (day 5) peak for two platelet-activating factors (PAF and TXB_2_) in the plasma of mice after SAH. Second, using two different experimental approaches to activate platelets, we established that activation of platelets after SAH worsened the early neurological outcome and caused higher DND incidence. Third, our study showed that platelet depletion in mice with SAH improved neurological outcomes and lowered DND incidence. Fourth, we evaluated inhibitors for five platelet receptor families involved in platelet activation and aggregation, and observed that tirofiban attenuated DND. Finally, in a translational experiment utilizing human SAH platelets, we demonstrated that inhibiting platelet aggregation, but not platelet activation, was capable of reducing platelet spreading. Overall, this study shows that platelets are key contributors to DND, but perhaps more importantly, we found that one specific mechanism is probably involved and identified that antagonism of GPIIb/IIIa is a potential treatment to prevent DND.

### Platelets cause microthrombosis and worse outcome after SAH

Several clinical studies have suggested that platelets may contribute to DND after SAH^6–12^, but ultimately there has not yet been a convincing and comprehensive study demonstrating platelets as a highly relevant therapeutic target to prevent DND. To this end, we designed a broad range of experiments to address this gap in knowledge. First, analyzing peripheral blood, we identified that PAF and TXB_2_, two important platelet activators, peak around day 5 post-SAH, which follows the timeline of DND (days 3–7 in mice^31^). In humans, PAF has also been reported to have a delayed peak occurring 5–9 days post-SAH, and PAF levels (on days 5–9) are significantly higher in patients experiencing DND (compared with patients without DND)^6,39^. Neither plasma thrombin nor PF4 were significantly elevated after SAH in mice. Thrombin, while elevated in the cerebrospinal fluid of patients SAH, is not elevated in the plasma after aneurysmal SAH^40^. In fact, it has even been suggested that plasma thrombin does not reflect the pathophysiology of SAH^40^. For PF4, there is very recent human evidence that PF4 is slightly elevated and associated with vasospasm^41^. Mechanistically, (pro)thrombin is released by the liver, so the release of prothrombin is independent of platelet activation. PAF and thromboxane A_2_ (the precursor of TXB_2_) are released by platelets, neutrophils and endothelial cells. As neutrophils are also activated by SAH, PAF and TXB_2_ are probably released by both cells, contributing to the overall high levels. On the other hand, PF4 is released primarily by platelets so there is not a cumulative effect by multiple cell types.

However, the increased PAF level on day 5 does not mean PAF causes DND. So next we asked: can exogenous PAF worsen outcomes and cause higher incidence of DND? Since platelets are known to be activated within hours of SAH, we first evaluated the response to PAF that is injected 1 h after SAH^42^. We used a dose of PAF that is known to promote platelet activation^43^. However, we found that this dose caused more than 90% mortality in SAH mice (12/13; Supplementary Figs. 2 and 3), which corresponded with increased formation of brain microthrombi (Supplementary Fig. 4). Thus, we reduced the dose of PAF by half. This lower dose reduced the mortality rate to 44.4% (16/36) in SAH mice receiving PAF compared with 8.3% (3/36) in SAH mice receiving saline. Our findings that PAF worsens outcome after SAH agrees with the study by Hirashima et al.^44^. In the next experiment, we administered PAF 3 days post-SAH to amplify the response to PAF during the second peak. Using this approach, we established that delayed PAF injection induces greater and more rapid onset of DND and delayed mortality, suggesting that delayed platelet activation in response to PAF can trigger DND. While PAF is primarily known for activation of platelets, it can also activate other cells, such as neutrophils^45^. Therefore it is possible that exogenous PAF may modify DND via platelet- or neutrophil-mediated mechanisms. While additional studies are needed to establish the relevance of platelet–neutrophil interactions in SAH pathogenesis, here we investigated if a specific platelet receptor(s) could prevent DND after SAH as we hypothesized that platelets are a therapeutic target.

To further confirm if platelet activation causes DND, we utilized a transgenic mouse (LIGHT^−/−^) that is reported to have platelet activation 3 days after wounding^23^. Interestingly, LIGHT^−/−^ mice had a higher DND incidence than WT mice after SAH (58.9% versus 30.0%). While we did not measure platelet activation after SAH, Dhall et al. reported that LIGHT^−/−^ mice had a delayed increase in P-selectin markers after wounding (days 3 and 7) and that the P-selectin level in LIGHT^−/−^ mice after wounding was significantly more than that of wounded C57BL/6 mice. Moreover, Dhall et al. observed that LIGHT^−/−^ mice were significantly more responsive to both ADP and a thromboxane A_2_ receptor agonist^23^. Thus, future experiments are needed to determine whether the higher DND incidence of LIGHT^−/−^ mice is due to SAH-induced increase of ADP or thromboxane A_2_. Collectively, our findings strongly suggest that platelets contribute to worse outcome and DND after SAH in mice.

### Microthrombi clustering

While SAH causes microthrombi that occlude microvessels of the brain, it is probably the phenomenon of microthrombi clustering that ultimately influences the development of DND and poor outcomes after SAH. An autopsy study of patients 3–14 days post-SAH observed microthrombi clusters occluding the brain vasculature and documented that the clusters corresponded with neuronal necrosis and regions of infarction^12^. In all the animals in this study, microthrombi were observed in 98.5% (264/268) of the brains counted. While in our mouse study no specific brain regions were more prone to microthrombi formation, we observed that microthrombi tended to cluster in brains with higher microthrombi counts, leaving some brain areas with low microthrombi counts (Supplementary Figs. 7–17 and Supplementary Table 4). Specifically, it is not the number of clusters, but rather the total amount of brain tissue affected by microthrombi clusters that is pathological. The correlational analysis of our data found a positive correlation between DND and both the microthrombi count (Spearman’s *ρ* = 0.480, *P* = 0.002) and the total microthrombi cluster area (Spearman’s *ρ* = 0.440, *P* = 0.015), but not between DND and cluster number (Spearman’s *ρ* = 0.182, *P* = 0.490) (Supplementary Table 6). Thus, our data, which agrees with the evidence from the human autopsy study by Stein et al.^12^, also suggests that microthrombi clustering is pathological (cluster area positively correlates with DND). Further evidence of a potential causal link between microthrombi clusters and DND is that the microthrombi cluster area was smaller (compared with injured controls) in mice depleted of platelets and in mice receiving tirofiban.

### Platelets are a therapeutic target for DND

As our data showed that platelet activation promotes DND, we then sought to determine whether platelet depletion before SAH would improve outcome and lessen DND. Using mice expressing the DTR selectively in platelets (PF4-DTR), we observed that mice depleted of platelets had significantly better neurological function, less microthrombi, and a lower incidence of DND than WT mice. It is important to stress that platelet depletion before SAH does not cause higher mortality or larger hematomas compared with nonplatelet-depleted SAH mice. This may be due to similar bleeding times in platelet-depleted versus non-depleted mice^24^.

Data from the platelet overactivation and depletion experiments indicate that platelets are important for promoting the development of DND after SAH, but whether platelets could represent a therapeutic target remained to be verified. When considering platelets as a focus for treatment, one needs to realize that platelets express several different surface receptors known to cause activation and have several unique signaling mechanisms responsible for degranulation and aggregation^46^. It is also known that platelets can become activated by any single activation receptor or by the contribution of several receptors, which each could be stimulated below the activation threshold^17,18^. Moreover, platelets can be activated even when inhibited^17^. Thus, the question about what to target on platelets is difficult. Many clinical studies on SAH have examined a variety of platelet antagonists with varied success. Here, we examined inhibitors of an aggregation receptor and four different platelet activation receptors, which have been reported to be associated with DND in patients with aneurysmal SAH patients^6,39,47^. In mice with SAH, targeting the PAF receptor, the thromboxane A_2_ receptor and GPIIb/IIIa was beneficial at preventing early neurological impairment in both females and males. These receptors have been suggested as therapeutic targets by others^37,48–52^. We then tested whether any of these antiplatelets could reduce DND incidence in SAH mice and found that while all three are beneficial in the early brain injury period, only the aggregation inhibitor, tirofiban, reduced DND. Daltroban showed a parallel reduction in both brain microthrombi count and DND compared with vehicle, although the reductions did not reach statistical significance (microthrombi, *P* = 0.086; DND, *P* = 0.159). The treatment benefit for GPIIb/IIIa inhibition might be greater than that of the activation receptor antagonists since the activation signaling pathways all converge on activation of GPIIb/IIIa and aggregation. Interesting, the iSPASM trial, which was a phase 1/2a trial examining tirofiban, reported that, in addition to being safe for use in patients with aneurysmal SAH, tirofiban had a lower incidence of DND than in placebo-treated patients; however, it was not powered for this outcome and thus a larger trial is needed^53^.

In this study, we observed a sex difference in treatment response to some antiplatelet drugs. While male mice benefited from all antiplatelets tested, female mice did not benefit from either P2Y or PAR-4 antagonists. It is well documented that platelets from females (human and mice) are inherently more reactive than males^54–57^. Documented reasons for female platelet hyperreactivity (compared with males) are higher sensitivity to agonists (such as ADP, collagen, and fibrinogen)^54,56^, higher P-selectin expression^55^, and higher levels of platelet-derived chemokines^57^. Estradiol has also been reported to increase platelet reactivity in females^58^. For PAR-4 antagonism, Leng et al. observed, in response to thrombin, greater reactivity of female mouse platelets^54^. This might be the reason for the observed lack of treatment effect of ML354 (specific PAR-4 antagonist) in female mice, and implies that independent studies should investigate whether a higher ML354 dose is needed in females to reach a therapeutic threshold. Female mice also benefit less from P2Y antagonists, which potentially might result from a reduced responsiveness to ADP (agonist of P2Y) in females^59,60^. The reduced activation of female platelets by ADP could suggest that P2Y antagonists are less effective in the binding/activation of the P2Y receptor in females or that a different dose of P2Y antagonists would be more effective. Regarding P2Y_12_ specifically, female patients have higher on-treatment effect for clopidogrel, meaning that a higher dose of clopidogrel is needed in female patients to have a similar reduction of platelet reactivity as male patients^61^. On the basis of documented sex differences in platelet reactivity and also response to antagonists, the sex-dependent dose-adjustment needs to be taken into account in clinical trials of antiplatelets.

### Clinical implications

There are many antiplatelet drugs available targeting a variety of platelet receptors, so choosing which antiplatelet drug to use for SAH is challenging. A 2007 meta-analysis of antiplatelet drugs found that there is a trend toward improved outcomes and less DND after SAH^13^, with a P2Y_12_ inhibitor (ticlopidine) showing the most promise^62^. However, ticlopidine has not been investigated further. Another P2Y_12_ antagonist, clopidogrel (plavix), has received more attention and has been examined in several clinical SAH studies. However, based on the outcome of these studies, clopidogrel does not seem to be effective at preventing DND nor improving outcome^14,63^. A more recent meta-analysis of 22 studies from multiple countries supports the 2007 meta-analysis that antiplatelet therapy can prevent DND and improve outcomes^14^. Although ticolopidine was not included in the recent meta-analysis, clopidogrel was, and the study found that while dual therapy of clopidogrel and aspirin could reduce angiographic vasospasm and in-hospital mortality, the dual therapy was not effective for reducing DND, symptomatic vasospasm, or hemorrhagic complications, and also did not improve outcomes^14^. With respect to P2Y antagonism, our study suggests that there may be a sex-specific benefit (male mice benefited, female mice did not) that needs to be considered when designing a clinical trial to study an inhibitor for this receptor; a sex-specific benefit for P2Y inhibitors might be a real concern for humans as well^64^.

If P2Y antagonism is not a treatment, then what is? The 2023 meta-analysis of trials with patients with SAH suggested that cilostazol (a phosphodiesterase-3 inhibitor) is beneficial in reducing DND, attenauting angiographic and symptomatic vasospasm, and improving outcomes^14^. While this meta-analysis only included studies from 2009 to 2018, older studies with phosphodiesterase-3 inhibitors have also suggested a therapeutic benefit^15,65^. In the current study, we did not examine a phosphodiesterase-3 inhibitor but a large randomized clinical trial for a phosphodiesterase-3 inhibitor is warranted.

Are there any other therapeutic targets on platelets? In our study, we found that although several antagonists of platelet activation provided an early benefit (that is, better day 1 neuroscore and fewer day 2 brain microthrombi), only the GPIIb/IIIa inhibitor reduced the incidence of DND. Although optimizing the treatment regimen of PAR inhibitors (dose, timing, or duration) may reduce DND, platelet activation occurs through multiple pathways that converge on GPIIb/IIIa-mediated aggregation, making GPIIb/IIIa an attractive therapeutic target. Moreover, our data also show that GPIIb/IIIa inhibition works on platelets from patients with SAH. As mentioned, the phase 1/2a iSPASM trial provided some evidence for the efficacy of GPIIb/IIIa antagonism^53^, but this needs to be evaluated in a multicenter clinical trial.

Overall, there is strong evidence that platelets are a therapeutic target to prevent DND with at least two targets (phosphodiesterase-3 inhibition and GPIIb/IIIa antagonism) being favored for testing in larger clinical trials. As antiplatelets carry a risk of hemorrhagic complications, care needs to be taken during clinical trials. So far, clinical evidence suggests that antiplatelets are safe for patients with SAH with no significantly higher risk of hemorrhagic complications^14,53^.

#### Comparison of platelets from humans and mice

While mice are widely used as the animal model for understanding platelet function, there are differences between mouse and human platelets that need to be mentioned. First, mice have about five times as many platelets as humans, and mice platelets are more reactive than human platelets^17,66–68^. Second, there are differences in expression of some receptors^46^. For example, mouse platelets lack PAR-1 but have PAR-3 (not expressed by human platelets)^69^. Regarding PARs, it is important to note that PAR antagonists that work in mice may not work in humans and vice versa^69^. Transcriptomic and proteomic analyses have highlighted differences in the intracellular signaling between mouse and human platelets^70,71^. In addition, it is important to note that, compared with human platelets, mouse platelets are more sensitive and responsive to some agonists, such as ADP and thrombin^68^. Regarding the aggregation receptor GPIIb/IIIa, there are subtle differences in the amino acids of the binding pocket, which reduce the effectiveness of GPIIb/IIIa antagonists in mice^29^. This is especially true for the antibodies and their fragments^29,72^. To this end, other laboratories have utilized humanized mouse models to confirm the utility of GPIIb/IIIa antagonists^29^. That said, tirofiban is reported to have similar inhibitory effects on both human and mouse platelets^72^.

### Limitations

No dose–response study was performed for any of the interventions as the dosages were adapted from studies of these drugs in other mice injury models. Also, it is important to note that administering daltroban and WEB2086 twice on the day of SAH was not sufficient to prevent DND; there is a possibility that daily doses of either of these antagonists could prevent DND, but this was not explored. Moreover, we did not test ML354 or the combination of A3P5PS and clopidogrel for their potential beneficial effects on DND since they were effective in only male mice in our 2-day study. While WEB2086 was effective at reducing microthrombi in the 2-day study, it was not effective for the day 7 brain microthrombi. A possible reason for this apparent discrepancy is that WEB2086 was only administered on day 0 and adding daily treatments may have a higher treatment effect. A contributing factor is also that PAF peaks on day 5, so WEB2086 given only on day 0 would not be effective to prevent the PAF receptor activation that occurs on day 5. Another limitation is that 25% ethanol, used as a vehicle for the platelet activation antagonists, can inhibit platelets, so these interventions might actually be dual antiplatelet therapy targeting two different antiplatelet pathways. While this limitation affects the platelet activation antagonists (A3P5PS and clopidogrel, daltroban, ML354, and WEB2086), saline was used as the vehicle for tirofiban, so our conclusion that tirofiban is a promising antiplatelet drug for preventing DND after SAH remains. For LIGHT^−/−^ mice, we did not measure platelet activation nor platelet-activating factors. Next, as only female mice were utilized in our 7-day study of platelet antagonists, further work needs to examine the therapeutic potential for antiplatelets to prevent DND in male mice, as well as in aged mice. We also need to perform a long-term study to determine whether the treatment effects are long lasting. For the exogenous stimulation study, PAF is known to also activate neutrophils, and neutrophils cause worse outcomes after SAH^73,74^, so there is a chance that neutrophils played a role in the worsened outcomes by PAF administration. Finally, humans have fewer platelets and less reactive platelets than mice, which may reduce the importance of platelets in humans with SAH. However, since the existing meta-analyses and individual clinical studies suggest that antiplatelets can improve outcome and reduce DND after SAH, the guidance provided by our animal study may play an important role during the design of clinical trials with platelet inhibitors for SAH.

## Conclusion

This comprehensive and comparative study highlights the critical role that platelets have in the pathogenesis of DND after SAH and suggests that platelets are an attractive therapeutic target for patients with SAH. While platelet depletion prevented DND in mice with SAH, platelet depletion is not clinically reasonable. Therefore, we examined six platelet antagonists and found that GPIIb/IIIa inhibition is more promising (than activation receptor antagonists) for preventing DND after SAH in mice and can also reduce platelet spreading in human SAH samples. We propose that it is time for antiplatelet drugs to be revisited in clinical trials for preventing DND after SAH.

## Supplementary information

Supplementary Information
