# Supplementary material for: Platelets cause microvascular occlusion and delayed neurological deficits after subarachnoid hemorrhage in mice
Source: Exp Mol Med. 2026 Apr 15;58(4):1242–53. doi: 10.1038/s12276-026-01696-1 (PMC13144347; doi:10.1038/s12276-026-01696-1)
Supplement: Supplementary file 1 — Supplementary Information [file 12276_2026_1696_MOESM1_ESM.pdf]

Supplementary Information
Platelets Cause Microvascular Occlusion and Delayed Neurological Deficits after Subarachnoid Hemorrhage in Mice

Ari Dienel,[1,*] Sung-Ha Hong,[1,*] Kiara Torres,[1] Kanako Matsumura,[1] Jose Guzman,[1] Peeyush Thankamani Pandit,[1] Bibek Samal,[1,2] Harveen Kaur,[1,3] Samitha Nemirajaiah,[1,3] Angelica Bernal,[1,4] H. Alex Choi,[1] Louise D. McCullough,[5] Spiros L. Blackburn,[1] Jaroslaw Aronowski,[5] Devin W. McBride[1,†]

[1] The Vivian L. Smith Department of Neurosurgery, McGovern Medical School, The University of Texas Health Science Center at Houston, Houston, Texas, USA
[2] Department of Biosciences, Rice University, Houston, Texas, USA
[3] Kinesiology Department, Rice University, Houston, Texas, USA
[4] Cornell University, Ithaca, New York, USA
[5] Department of Neurology, McGovern Medical School, The University of Texas Health Science Center at Houston, Houston, Texas, USA

* Each author contributed equally

†Corresponding author:
Devin W. McBride, PhD, devin.w.mcbride@uth.tmc.edu

Supplementary Methods
<u>Preliminary Study</u>
We ran an initial study to determine the regimen of PAF. We started with a dose of 19 μg/kg PAF intravenously injected. Two a 2-day study, 4 sham females and 9 SAH mice (2 males, 7 females) were administered PAF 1 hr after SAH. For the 7-day study, 8 sham and 3 SAH female mice were administered PAF 3 days after SAH. Based on the results, we chose to perform a dose of 9.5 μg/kg PAF intravenously injected in the main study.

<u>Main Study</u>
<u>Delayed neurological deficits:</u> Delayed neurological deficits (DND) is one component of delayed cerebral ischemia pathology.[1] Specifically, DND is characterized as a delayed decline in neurological behavior and is observed in humans[1] and mice.[2] In a previous paper, we characterized DND in mice as follows: 1) mice must have some recovery of behavioral performance (using neuroscore in our study) from the neuroscore on day 1; 2) after some recovery, mice experiencing a neuroscore which is 5 or more points less than their best performance (from any prior post-SAH day).[2] Animals experiencing delayed death (after some functional recovery) were also considered as developing DND since their neuroscore would be equal to 0. Supplementary Table 1 displays some examples of mice experiencing DND and those not. Time to DND was analyzed by a log-rank test. DND incidence was calculated as

$$\text{DND Incidence (\%)} = \frac{\begin{pmatrix}\text{\% of animals that experienced delayed behavioral} \\ \text{decline in the neuroscore test of 5 points or more}\end{pmatrix}}{(\text{total number of animals surviving more than 2 days})} x100$$

Supplementary Table 1. Neuroscore data from selected mice to display how DND was identified.

Mouse ID	Group	Neuroscore							Developed DND?
		D1	D2	D3	D4	D5	D6	D7	
4286-11	SAH+Daltroban	22	21	24	23	N/A	N/A	N/A	Yes – died on D5
2925	SAH+Saline	15	22	24	16	10	5	2	Yes on D4 (24->16)
6190-2	SAH+Tirofiban	21	24	23	21	N/A	N/A	N/A	Yes – died on D5
6191-1	SAH+50%EtOH	19	21	21	17	12	20	22	Yes on D5 (21->12)
1015	SAH+Saline	13	19	20	11	24	24	24	Yes on D3 (20->11)
6190-1	SAH+Tirofiban	20	24	24	23	24	22	23	No – behavior did not decline by 5 or more points
4286-2	SAH+WEB2086	23	13	N/A	N/A	N/A	N/A	N/A	No – died on D3 but did not have any behavioral recovery
3437	SAH+Tirofiban	23	23	24	24	24	24	24	No – no behavioral decline or death

Microthrombi Counting: Microthrombi are counted after staining the brain slices with MSB. In Supplementary Fig. 1, an example is shown for microthrombi counting.

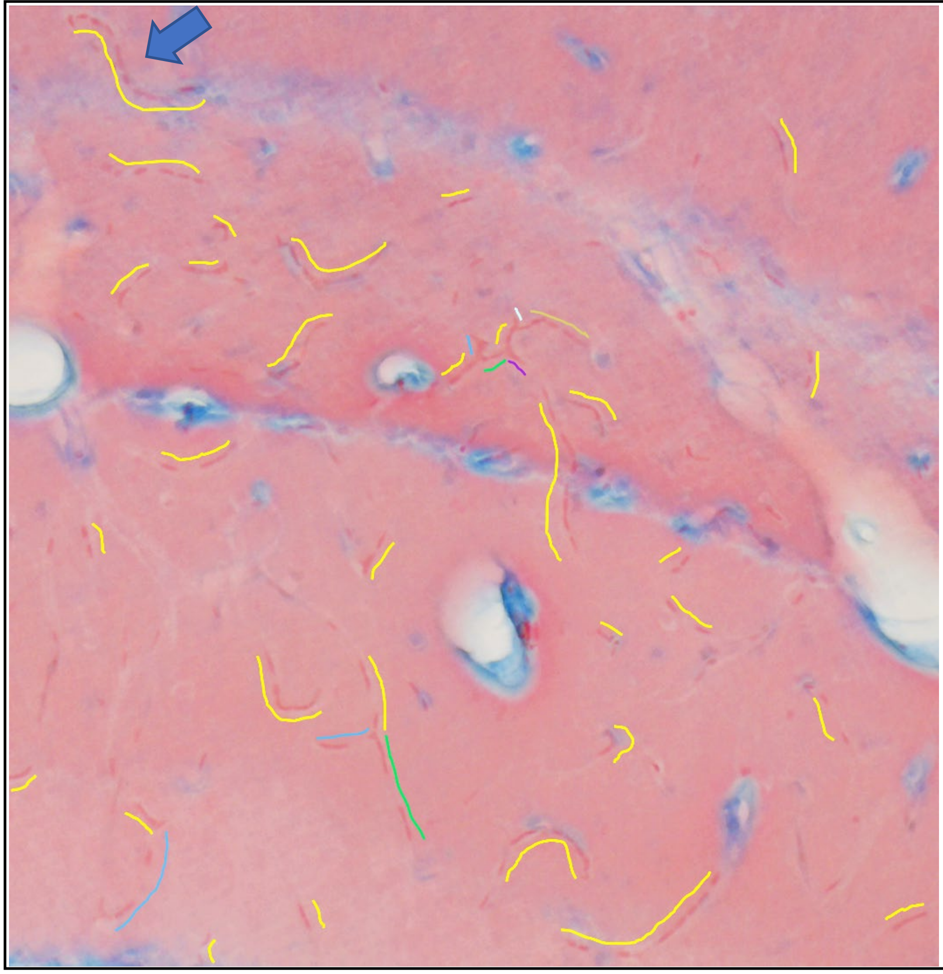

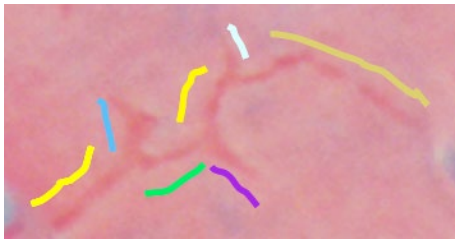

Supplementary Fig. 1. Representative MSB stained brain slice. Microthrombi appear in red. Each line segment drawn is counted as a single microthrombi. In vessels which branch, each branch is counted as one microthrombi. For example, the zoomed portion on the top right shows microthrombi occluded a branched vessel. This is counted as seven microthrombi since they are seven vessel segments that are occluded. Other vessels have segmented microthrombi (arrow in left figure) but this is counted as a single microthrombi.

Supplementary Results

Preliminary Study

In the 2-day study using 19 μg/kg PAF, 0/4 sham, and 8/9 SAH mice died. The SAH mice died within 2 hours after injection (3 died within 20 minutes, 3 died at about 30 minutes, 1 died at 1 hour, and 1 died at 110 minutes) (Supplementary Fig. 2). In the 7-day study using 19 μg/kg PAF, 3/8 sham mice and 4/4 SAH mice died within 30 minutes after injection (Supplementary Fig. 3). Based on our findings, we reduced the PAF dose by half to reduce the substantial immediate mortality from PAF.

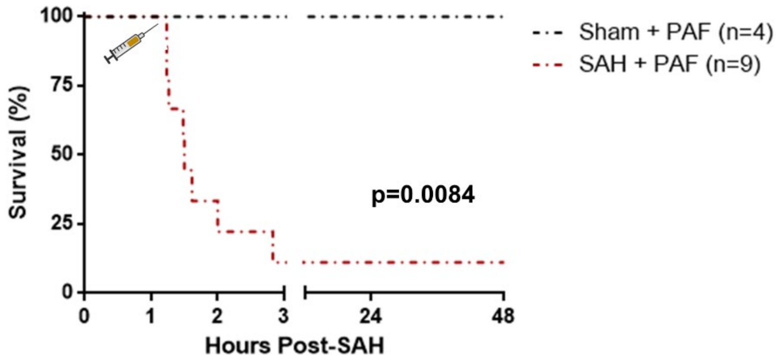

Supplementary Fig. 2. Kaplan Meir curve of mice given 19 μg/kg PAF 1 hr post-SAH.

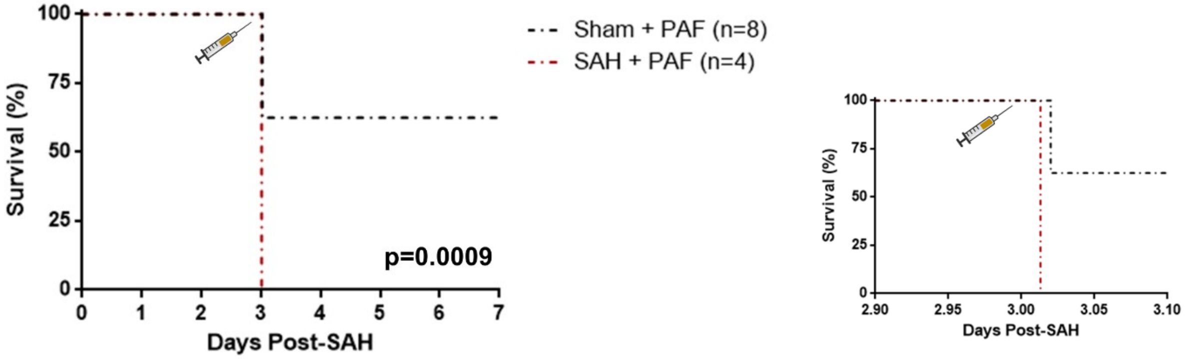

Supplementary Fig. 3. Survival curve of mice injected with 19 μg/kg PAF 3 days post-SAH. Right graph is zoomed onto day 3 to show all deaths were within 30 minutes after injection.

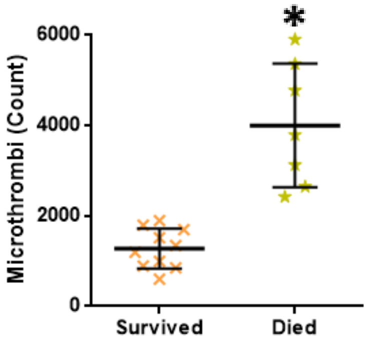

Supplementary Fig. 4. Mice dying from PAF injection have significantly more microthrombi than mice surviving from PAF administration. * $p<0.05$.

Main Study – Animals

Exclusion Rates

Mice were excluded or replaced for the following groups and reasons. The data from mice that were replaced were not included in any part of this study, including the final animal counts reported in the main manuscript.

Serum markers of platelet activators over time – Since we needed serum to analyze the markers of platelet activating factors, we required animals to survive until the chosen endpoint (1, 3, 5, or 7 days post-SAH). One mouse died and was replaced for the day 1 and 3 groups. Two mice died and were replaced in the day 5 group. Three mice died and were replaced for the day 7 group.

Platelet activation by PAF 2-day study – One male SAH + Saline (ICP<60 mmHg) and one male SAH + PAF (ICP>100 mmHg) were excluded and replaced. Two female SAH + Saline and one female SAH + PAF were excluded and replaced for having ICP greater than 100 mmHg.

Platelet activation by PAF 7-day study – Four SAH + Saline and three SAH + PAF mice were excluded and replaced due to ICP less than 60 mmHg. Three SAH + Saline and two SAH + PAF mice were excluded and replaced due to ICP greater than 100 mmHg. Three SAH + Saline and five SAH + PAF mice were excluded and replaced because they died before day 3. One SAH + Saline and one SAH + PAF mouse were excluded and replaced due to complications during surgery.

Platelet hyperactive (LIGHT-/- mouse) study – One male SAH WT, three male SAH LIGHT-/-, and two female SAH LIGHT-/- mice were excluded and replaced because they died before day 1 behavior. One male SAH WT, one male SAH LIGHT-/-, and one female SAH LIGHT-/- mice were excluded and replaced due to ICP greater than 100 mmHg. Two male SAH WT, four male SAH LIGHT-/-, two female SAH WT, and one female SAH LIGHT-/- mice were excluded and replaced due to ICP less than 60 mmHg. Three female SAH LIGHT-/- mice were excluded from DND analysis since they failed to have any neuroscore recovery after day 1.

Platelet depletion 2-day study – One male PF4-DTR SAH + DT and one female WT SAH + DT mice were excluded and replaced because neuroscore was less than 8 on day 1. Two male PF4-DTR SAH + DT mice were excluded and replaced since ICP was less than 60 mmHg. Two male PF4-DTR SAH + DT mice were excluded and replaced due to failed platelet depletion. One female

PF4-DTR Sham + DT and one female PF4-DTR SAH + DT mouse were excluded from platelet counts due to issues with clotting and processing prior to platelet counting.

Platelet depletion 7-day study – One male WT SAH + DT, one male PF4-DTR SAH + DT, and one female WT SAH + DT mice were excluded and replaced because they died immediately following SAH induction. One female PF4-DTR SAH + DT mouse was excluded and replaced because it died before day 1 behavior. One male WT SAH + DT, one female WT SAH + DT, and one female PF4-DTR SAH + DT mice were excluded and replaced because neuroscore was less than 8 on day 1 (a priori exclusion criteria). Six male WT SAH + DT, two male PF4-DTR SAH + DT, one female WT SAH + DT, and three female PF4-DTR SAH + DT mice were excluded and replaced since ICP was less than 60 mmHg. One female WT SAH + DT and two female PF4-DTR SAH + DT mice were excluded and replaced since ICP was greater than 100 mmHg. Two female WT SAH + DT mice were excluded from DND analysis since they failed to have any neuroscore recovery after day 1. Three male WT SAH + DT, three male PF4-DTR SAH + DT, and six female PF4-DTR SAH + DT mouse were excluded from platelet counts due to issues with clotting and processing prior to platelet counting. Five male WT SAH + DT, two male PF4-DTR SAH + DT, eleven female WT SAH + DT, and two female PF4-DTR SAH + DT mice were excluded from platelet counts due to dying before day 7 euthanasia.

Platelet antagonism 2-day study – Two male SAH + Saline, one male SAH + 50% EtOH, one male SAH + Daltroban, two female SAH + Saline, and one female SAH + 50% EtOH mice were excluded and replaced due to ICP less than 60 mmHg. Two male SAH + 50% EtOH, one male SAH +Tirofiban, one female SAH + Saline, and one female SAH + Daltroban mice were excluded and replaced due to ICP greater than 100 mmHg. One male SAH + A3P5PS + Clopidogrel, one male SAH +Tirofiban, and one female SAH + 50% EtOH mice were excluded and replaced because neuroscore was less than 8 on day 1 (a priori exclusion criteria). One male SAH + Daltroban, two male SAH + WEB2086, two female SAH + A3P5PS + Clopidogrel, one female SAH + Daltroban, two female SAH + ML354, two female SAH + WEB2086, and two female SAH + Tirofiban mice were excluded and replaced because they died immediately following SAH induction.

Platelet antagonism 7-day study (females only) – One SAH + Tirofiban mouse was excluded and replaced due to ICP less than 60 mmHg. One SAH + 50% EtOH, six SAH + Daltroban, and two SAH + Tirofiban mice were excluded and replaced due to ICP greater than 100 mmHg. One SAH + Saline, and one SAH + WEB2086 mice was excluded and replaced because neuroscore was less than 8 on day 1 (a priori exclusion criteria). Five SAH + 50% EtOH, and two SAH + Tirofiban mice were excluded and replaced because they died immediately following SAH induction. One SAH + 50% EtOH, one SAH + 50% EtOH, one SAH + WEB2086, and one SAH + Tirofiban mice were excluded and replaced because they did not survive to behavioral testing on day 1 (a priori exclusion criteria for the 7-day study). One SAH + Saline, one SAH + 50% EtOH, and one SAH + WEB2086 mice were excluded from DND analysis since they failed to have any neuroscore recovery after day 1.

<u>Morality Rates</u>

The mortality rates for this study are as follows: 0/10 male Sham, 0/24 female Sham, 7/24 female SAH, 2/32 male SAH + Saline, 11/52 female SAH + Saline, 1/7 male Sham + PAF, 1/17 female Sham + PAF, 11/20 male SAH + PAF, 20/44 female SAH + PAF, 0/7 LIGHT$^{-/-}$ male Sham, 0/7

LIGHT$^{-/-}$ female Sham, 9/29 LIGHT$^{-/-}$ male SAH, 13/30 LIGHT$^{-/-}$ female SAH, 0/15 male WT Sham + DT, 0/15 female WT Sham + DT, 5/40 male WT SAH + DT, 12/40 female WT SAH + DT, 0/7 male PF4-DTR Sham + DT, 0/7 female PF4-DTR Sham + DT, 3/40 male PF4-DTR SAH + DT, 3/38 female PF4-DTR SAH + DT, 6/30 female SAH + 50% EtOH, 1/10 male SAH + 50% EtOH, 0/10 female SAH + A3P5P + Clopidogrel, 0/10 male SAH + A3P5P + Clopidogrel, 5/30 female SAH + Daltroban, 0/10 male SAH + Daltroban, 1/10 female SAH + ML354, 0/10 male SAH + ML354, 6/30 female SAH + WEB2086, 0/10 male SAH + WEB2086, 3/30 female SAH + Tirofiban, and 0/10 male SAH + Tirofiban.

Other Results

We tested for correlations between the various platelet activating factors (Supplementary Table 2).

For the study examining platelet activation by exogenous PAF, mice tended to expire quickly after PAF injection. The mortality times are documented in Supplementary Table 3. In mice surviving from PAF administration, the brain microthrombi counts were significantly lower than that of mice dying from PAF injection (Supplementary Fig. 4).

For the platelet depletion study, platelets were counted using Hemavet analysis of platelet-rich plasma (Supplementary Fig. 5 and 6).

Whole brain images of MSB stained -2 from Bregma brain slices are displayed in Supplementary Fig. 7 and 11. Clusters of microthrombi were identified by areas in which more than 10 microvessels were occluded by microthrombi in an area of 70,000μm^2 (*i.e.* circle with a radius of 150μm). In areas where the clusters overlapped, the clusters were considered to be part of the same cluster (see Supplementary Fig. 8). Correlation between microthrombi counts or cluster area and neuroscore or DND or infarct area are presented in Supplementary Tables 4-6.

For LIGHT$^{-/-}$ mice, there are no statistically significant sex differences for either outcome. For neuroscore, there is no statistically significant difference between neuroscore for male vs female LIGHT$^{-/-}$ mice with SAH (repeated measures two-way ANOVA on ranks followed by Mann-Whitney U tests for each day, p=0.081-0.931 for the intergroup comparisons). Regarding DND incidence, there is also no significant difference between male and female LIGHT$^{-/-}$ SAH mice (p=0.1432).

Demographics of the humans included into the platelet spreading experiments are documented in Supplementary Table 7. Of the seventeen SAH patients, five patients have samples included in both the early (days 1-2) and delayed (days 4-10) timepoints. On days 1-2 post-SAH, 11 patients were included with samples collected on either day 1 or day 2. All 11 patients were used for saline, 7 patients were used for tirofiban, and 4 patients were used for A3P5PS. For days 4-10 post-SAH, 11 patients were included with 3 patients having samples collected on day 4, 7, and 10, 1 patient had samples collected on days 4 and 7, 1 patient had samples collected on days 4 and 10, 2 patients had samples collected on days 7 and 10, and 4 patients only had one sample collected during the delayed time point (Supplementary Table 8).

Statistical reports for all data are presented in Supplementary Tables 9-26.

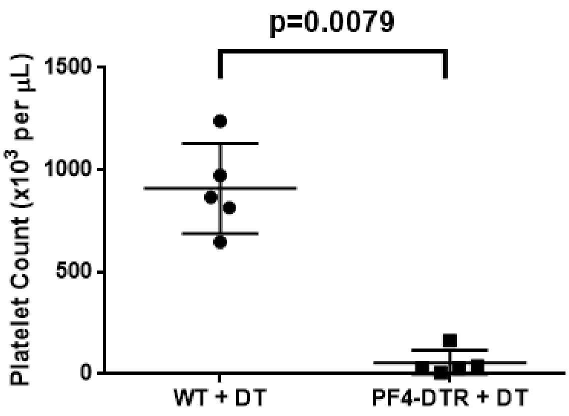

Supplementary Fig. 5. Platelet counts for naïve mice administered diphtheria toxin (DT). Mice were euthanized 1 day after 3 doses on every other day. WT: wild-type mice.

Supplementary Table 2. Correlational Analysis for Plasma Platelet Activating Factors. Pearson correlation analysis.

Sham		**PAF vs Thrombin<br>ρ=0.840, p=0.036**	PAF vs TXB_2<br>ρ=0.687, p=0.132	PAF vs PF4<br>ρ=0.701, p=0.121
		Thrombin vs TXB_2<br>ρ=0.464, p=0.355	Thrombin vs PF4<br>ρ=0.237, p=0.652	TXB_2 vs PF4<br>ρ=0.763, p=0.078
SAH	Day 1	PAF vs Thrombin<br>ρ=-0.579, p=0.229	PAF vs TXB_2<br>ρ=0.170, p=0.748	PAF vs PF4<br>ρ=-0.475, p=0.341
		Thrombin vs TXB_2<br>ρ=0.058, p=0.912	Thrombin vs PF4<br>ρ=0.132, p=0.803	TXB_2 vs PF4<br>ρ=0.562, p=0.245
	Day 3	PAF vs Thrombin<br>ρ=0.535, p=0.274	PAF vs TXB_2<br>ρ=-0.292, p=0.574	PAF vs PF4<br>ρ=-0.071, p=0.894
		Thrombin vs TXB_2<br>ρ=-0.529, p=0.274	Thrombin vs PF4<br>ρ=-0.745, p=0.089	TXB_2 vs PF4<br>ρ=0.606, p=0.202
	Day 5	PAF vs Thrombin<br>ρ=-0.099, p=0.853	PAF vs TXB_2<br>ρ=-0.754, p=0.083	PAF vs PF4<br>ρ=0.576, p=0.232
		Thrombin vs TXB_2<br>ρ=0.417, p=0.411	Thrombin vs PF4<br>ρ=0.241, p=0.645	TXB_2 vs PF4<br>ρ=-0.569, p=0.239
	Day 7	PAF vs Thrombin<br>ρ=0.124, p=0.815	PAF vs TXB_2<br>ρ=0.463, p=0.355	PAF vs PF4<br>ρ=-0.114, p=0.829
		Thrombin vs TXB_2<br>ρ=-0.391, p=0.444	Thrombin vs PF4<br>ρ=0.009, p=0.987	TXB_2 vs PF4<br>ρ=0.445, p=0.377

Supplementary Table 3. Mortality times post-injection of PAF or Saline. For the 2-Day Study, injection occurred 1 hr post-SAH. For the 7-Day Study, injection occurred 3 days post-SAH.

		Mouse ID	Group	Notes
2-Day Study	Female	F4	SAH+PAF	Died 15 min post-PAF injection
		F5	SAH+PAF	Died 40 min post-PAF injection
		2149	SAH+PAF	Died 15 min post-PAF injection
		2323	SAH+PAF	Died 1.5 hours post-PAF injection
		3499-3	SAH+PAF	Died 30 min post-PAF injection
		3499-4	SAH+PAF	Died 10 min post-PAF injection
		3499-5	SAH+PAF	Died 10 min post-PAF injection
		3143-20	SAH+Saline	Died 14 hrs post-Saline injection
	Male	2265	Sham+PAF	Died 10 min post-PAF injection
		2247	SAH+PAF	Died 1 hour post-PAF injection
		2248	SAH+PAF	Died 1 hour post-PAF injection
		2730	SAH+PAF	Died 1 hour post-PAF injection
		3547-9	SAH+PAF	Died 30 min post-PAF injection
		4454	SAH+PAF	Died 35 min post-PAF injection
		4462	SAH+PAF	Died 15 min post-PAF injection
		4463	SAH+PAF	Died 23 min post-PAF injection
		4464	SAH+PAF	Died 49 min post-PAF injection
		4468	SAH+PAF	Died 30 min post-PAF injection
		4481	SAH+PAF	Died 30 min post-PAF injection
		4482	SAH+PAF	Died 30 min post-PAF injection
		3433	SAH+Saline	Died 40 min post-Saline injection
		3531	SAH+Saline	Died 17 hrs post-Saline injection
7-Day Study	Female	3143-11	Sham+PAF	Died 1 day post-PAF injection
		3143-7	SAH+PAF	Died 6 days post-PAF injection
		3143-12	SAH+PAF	Died 4 days post-PAF injection
		3143-16	SAH+PAF	Died 4 days post-PAF injection
		3143-18	SAH+PAF	Died 4 days post-PAF injection
		3143-33	SAH+PAF	Died 7 days post-PAF injection
		3143-34	SAH+PAF	Died 1 hr post-PAF injection
		3143-35	SAH+PAF	Died 6 days post-PAF injection
		3143-85	SAH+PAF	Died 1 hr post-PAF injection
		3569-1	SAH+PAF	Died 12-16 hrs post-PAF injection
		3569-7	SAH+PAF	Died 20 min post-PAF injection
		3569-1	SAH+PAF	Died 20 min post-PAF injection
		4122	SAH+PAF	Died 30 min post-PAF injection
		4123	SAH+PAF	Died 4 hrs post-PAF injection
		3687	SAH+Saline	Died 6 days post-PAF injection
		3143-52	SAH+Saline	Died 5 days post-PAF injection
		3143-67	SAH+Saline	Died 7 days post-PAF injection

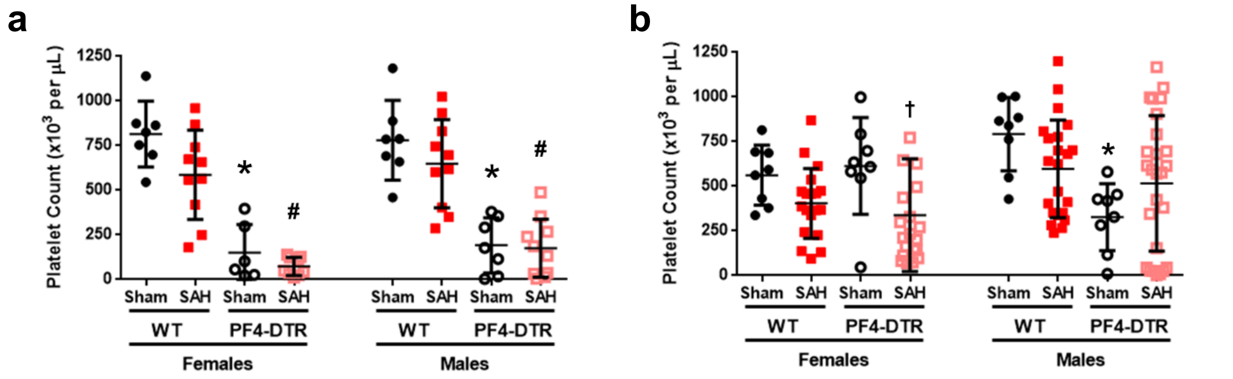

Supplementary Fig. 6. Platelet counts for the 2-day (**a**) and 7-day study (**b**). ***** p<0.05 vs WT Sham, **#** p<0.05 vs WT SAH, **†** p<0.05 vs (PF4-DTR) Sham. WT: wild-type mice.

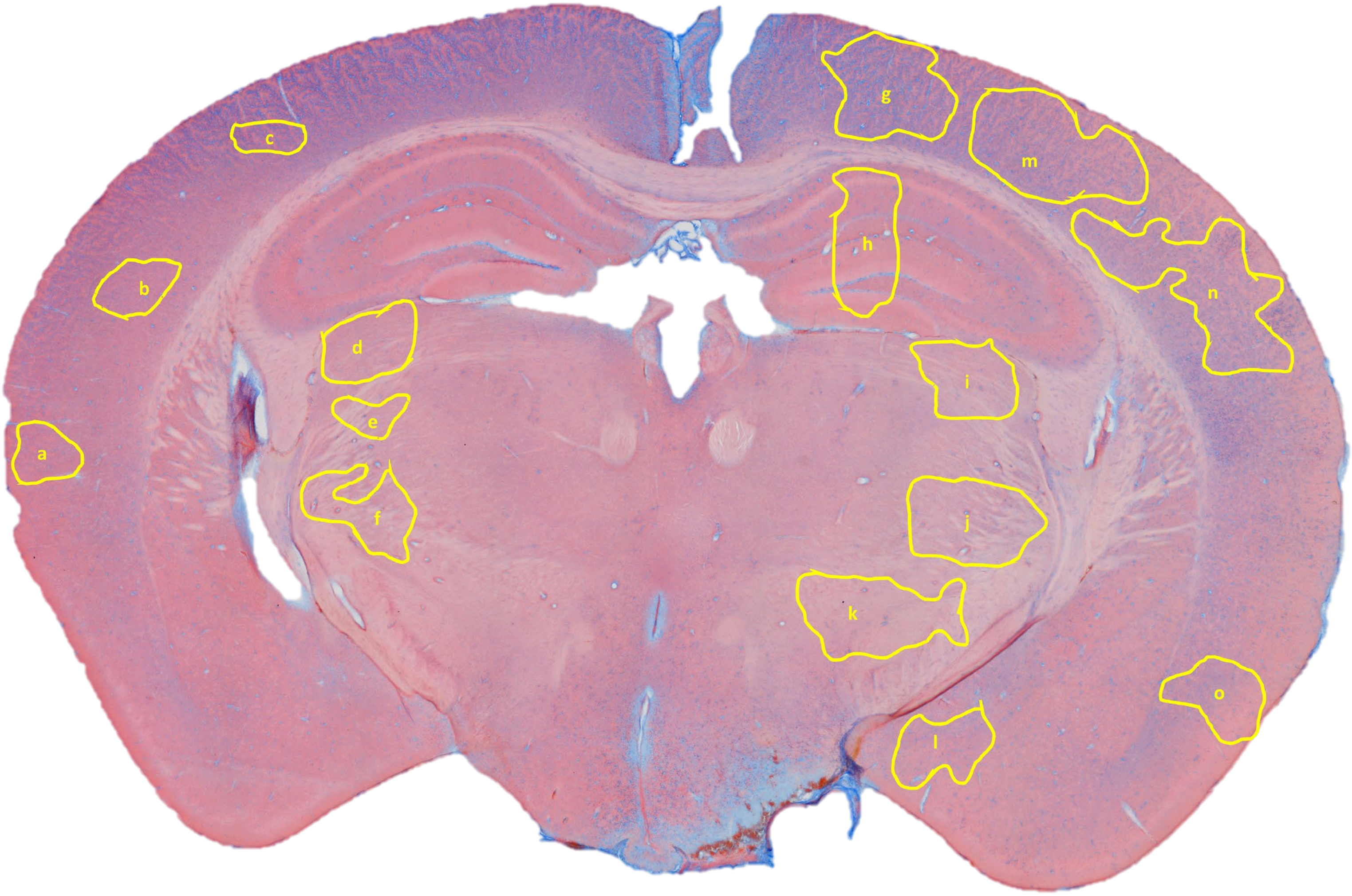

Supplementary Fig. 7. Representative SAH brain stained with MSB for microthrombi; clusters are outlined in yellow. Cluster i dentification and quantification is shown in Supplementary Fig. 8. Each cluster is zoomed in on Supplementary Fig. 9-10.

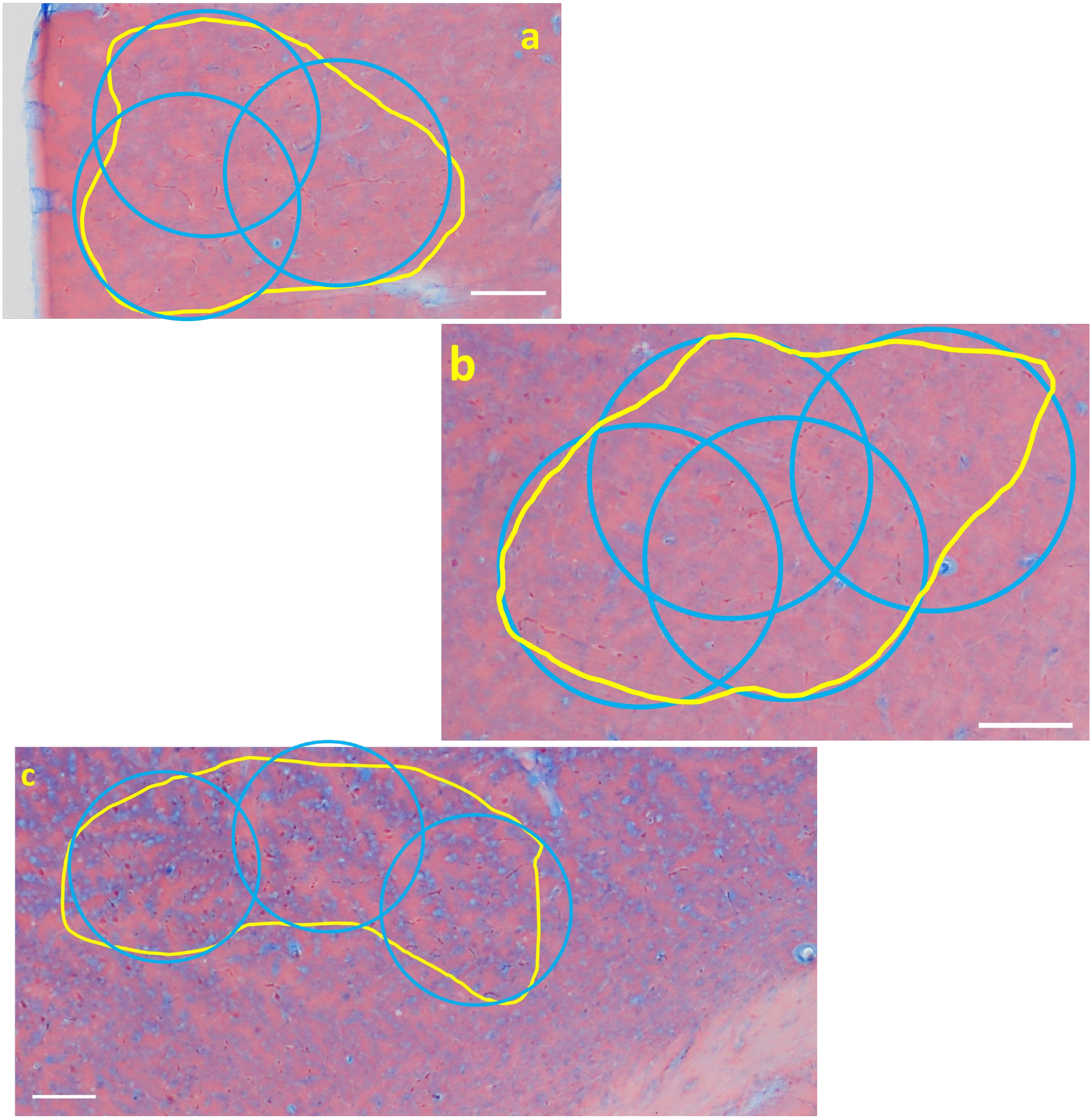

Supplementary Fig. 8. Zoomed in MSB-stained SAH brain clusters a-c in Supplementary Fig. 7. Total cluster is outlined in yellow. Clusters were identified as an area of 70,000μm^2 (e.g. circle with radius of 150μm, blue circles) having more than 10 microthrombi. Overlapping blue circles were combined to form the real cluster (yellow outline). Images are sharpened for better visualization of microthrombi. Bar = 100μm.

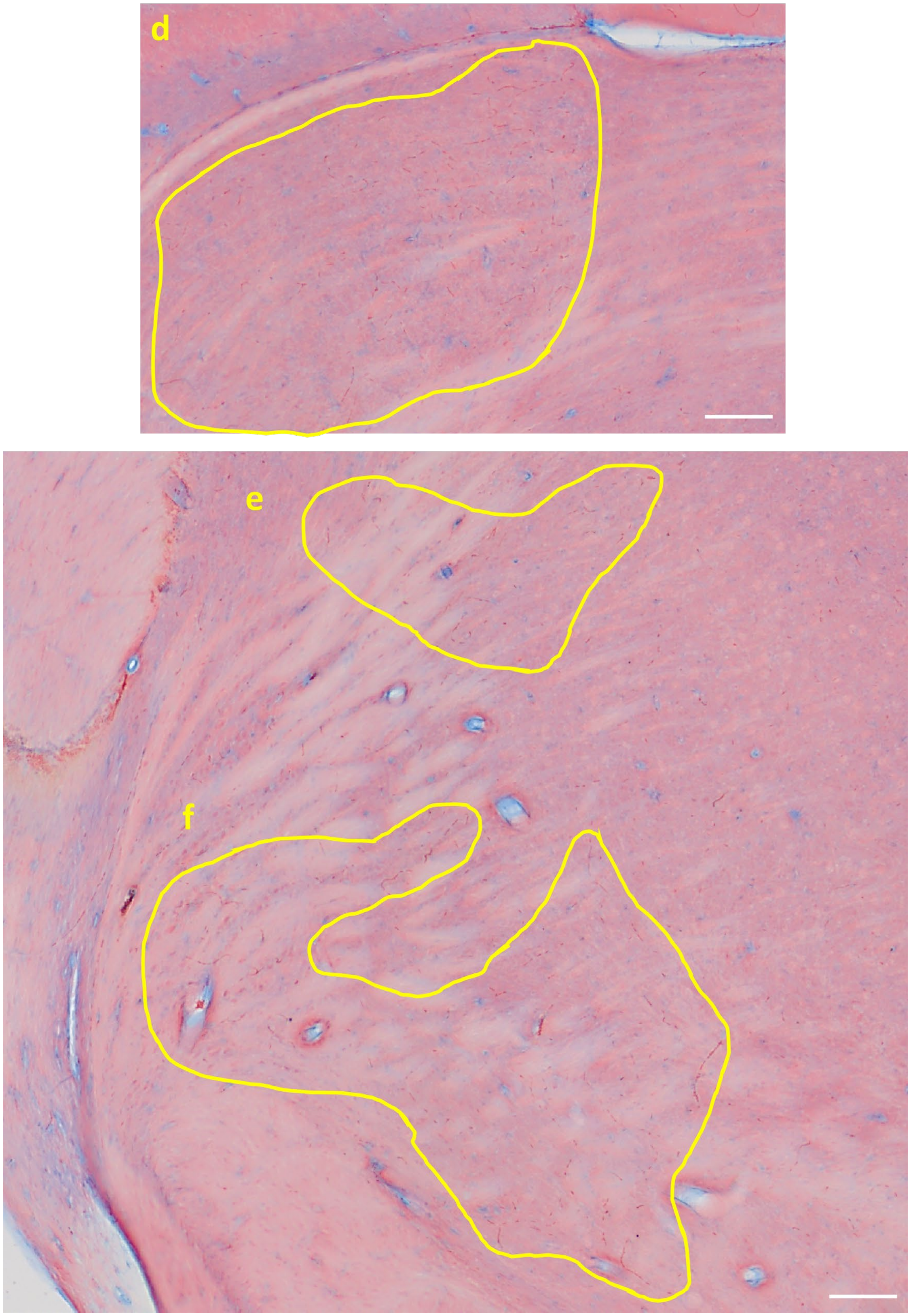

Supplementary Fig. 9. Zoomed in MSB-stained SAH brain clusters d-f in Supplementary Fig. 7. Clusters are outlined in yellow. Images are sharpened for better visualization of microthrombi. Bar = 100μm.

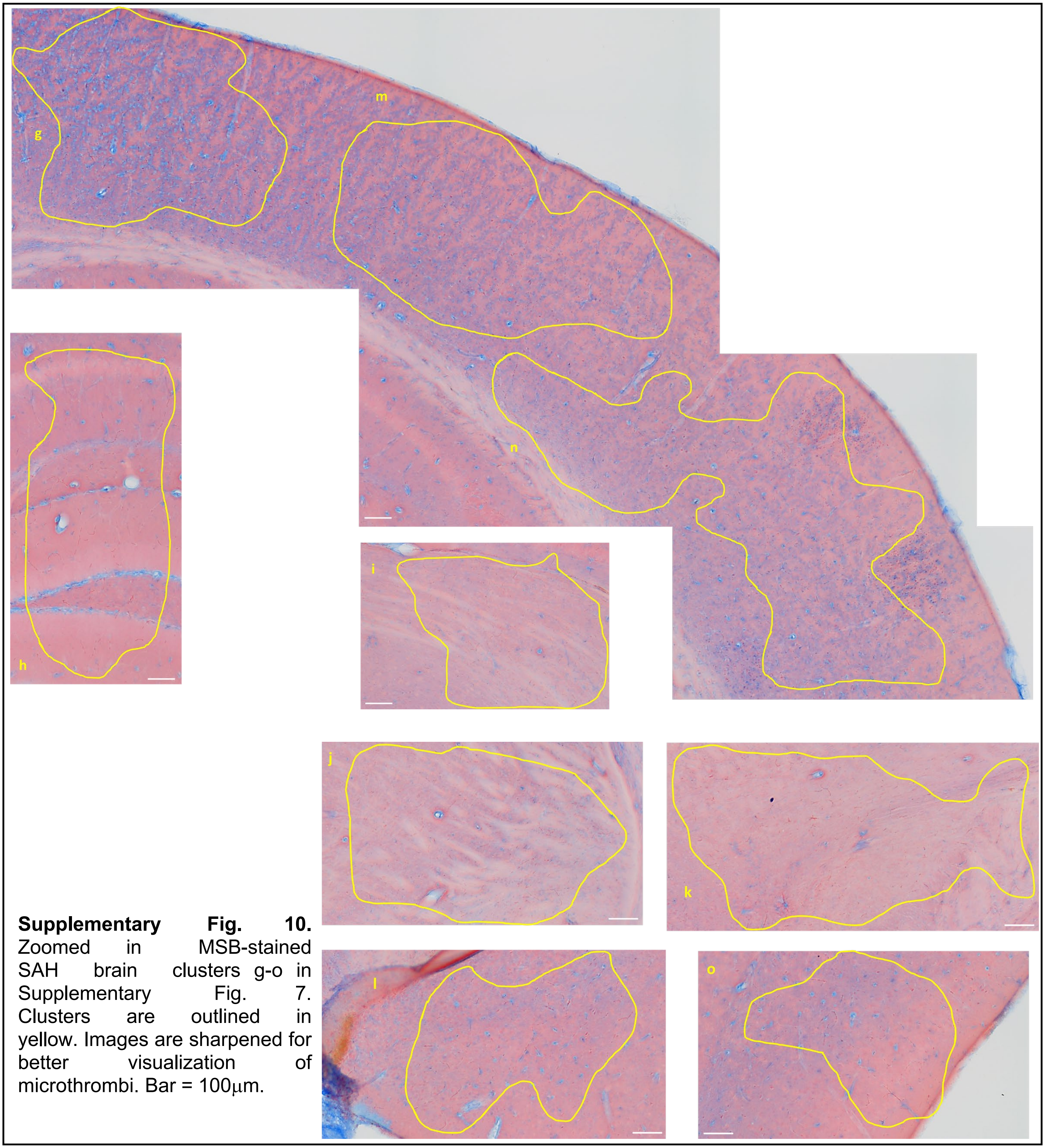

Supplementary Fig. 10. Zoomed in MSB-stained SAH brain clusters g-o in Supplementary Fig. 7. Clusters are outlined in yellow. Images are sharpened for better visualization of microthrombi. Bar = 100μm.

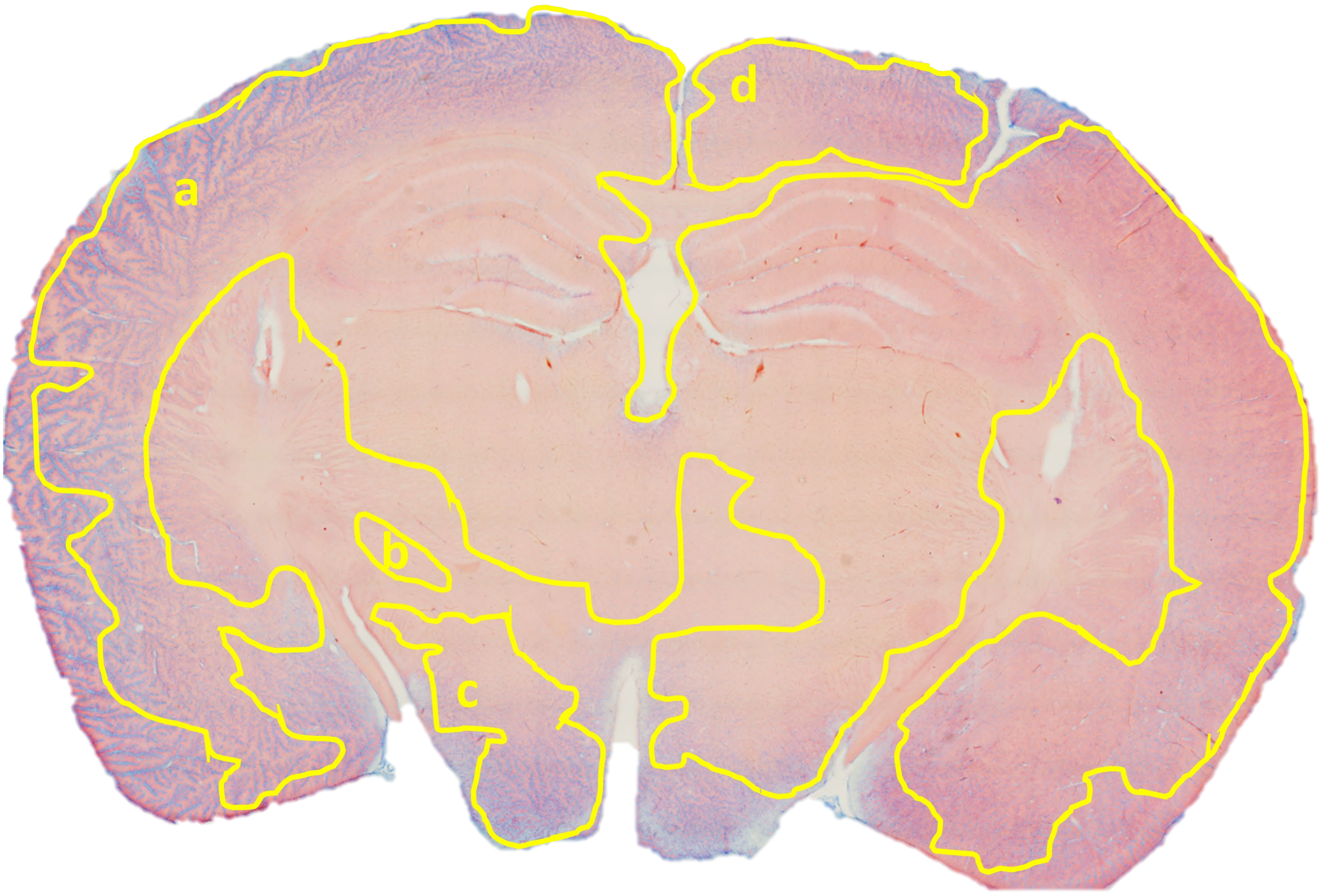

Supplementary Fig. 11. Representative SAH brain stained with MSB for microthrombi; clusters are outlined in yellow. Each cluster is zoomed in on Supplementary Fig. 12-17.

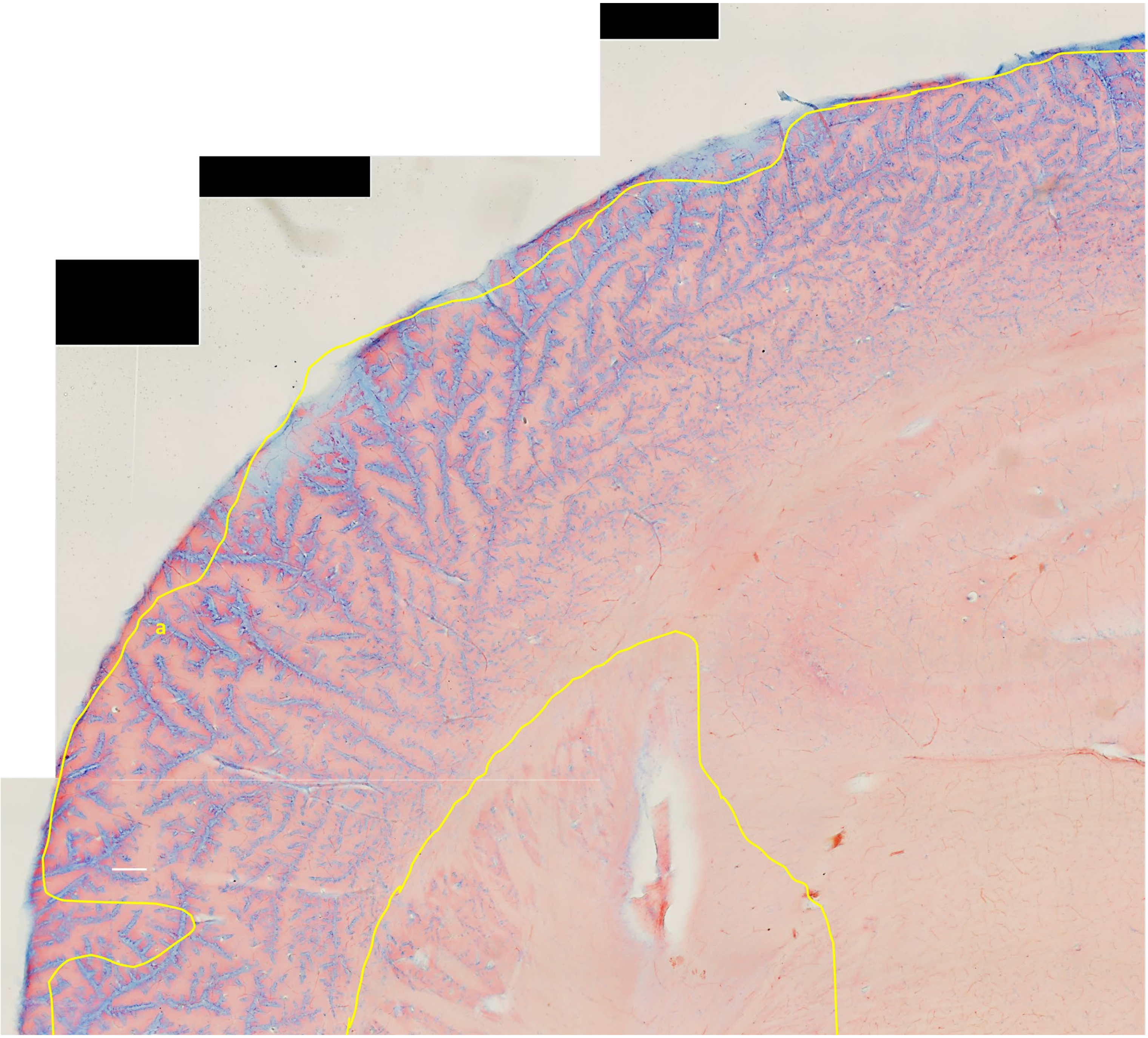

Supplementary Fig. 12. Zoomed in MSB-stained SAH brain clusters a in Supplementary Fig. 11. Clusters are outlined in yellow. Images are sharpened for better visualization of microthrombi. Bar = 100μm.

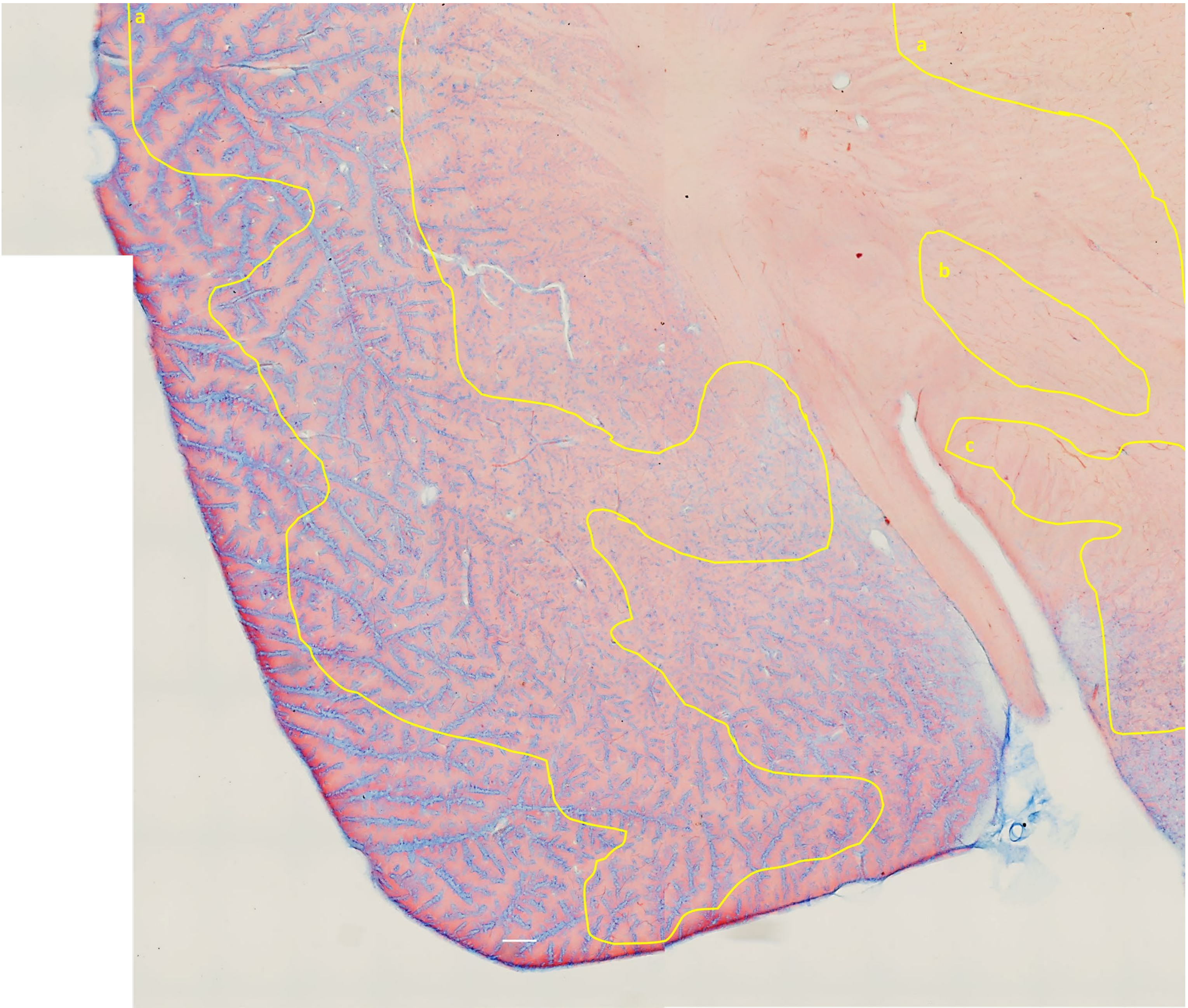

Supplementary Fig. 13. Zoomed in MSB-stained SAH brain clusters a-c in Supplementary Fig. 11. Clusters are outlined in yellow. Images are sharpened for better visualization of microthrombi. Bar = 100μm.

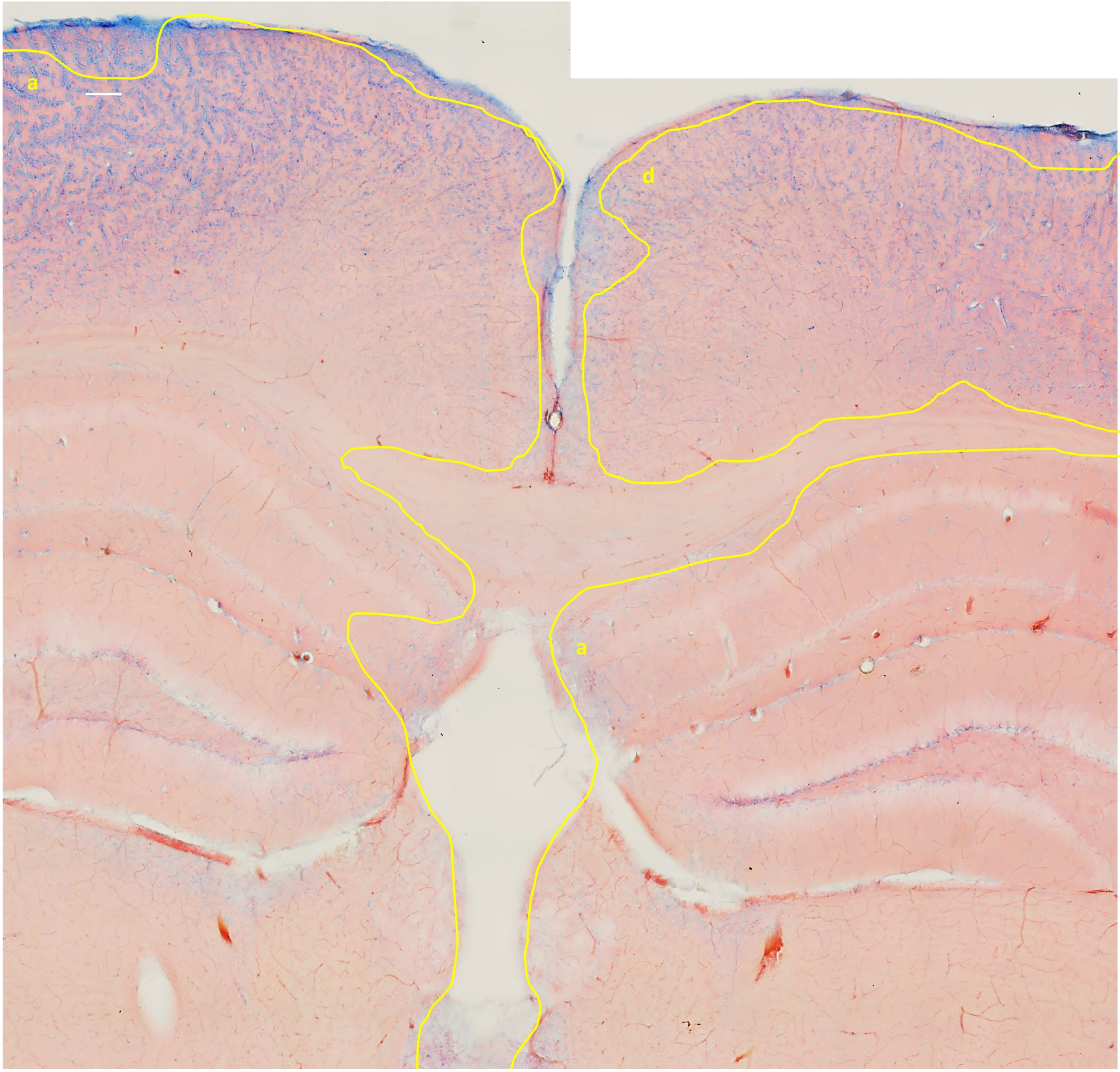

Supplementary Fig. 14. Zoomed in MSB-stained SAH brain clusters a and d in Supplementary Fig. 11. Clusters are outlined in yellow. Images are sharpened for better visualization of microthrombi. Bar = 100μm.

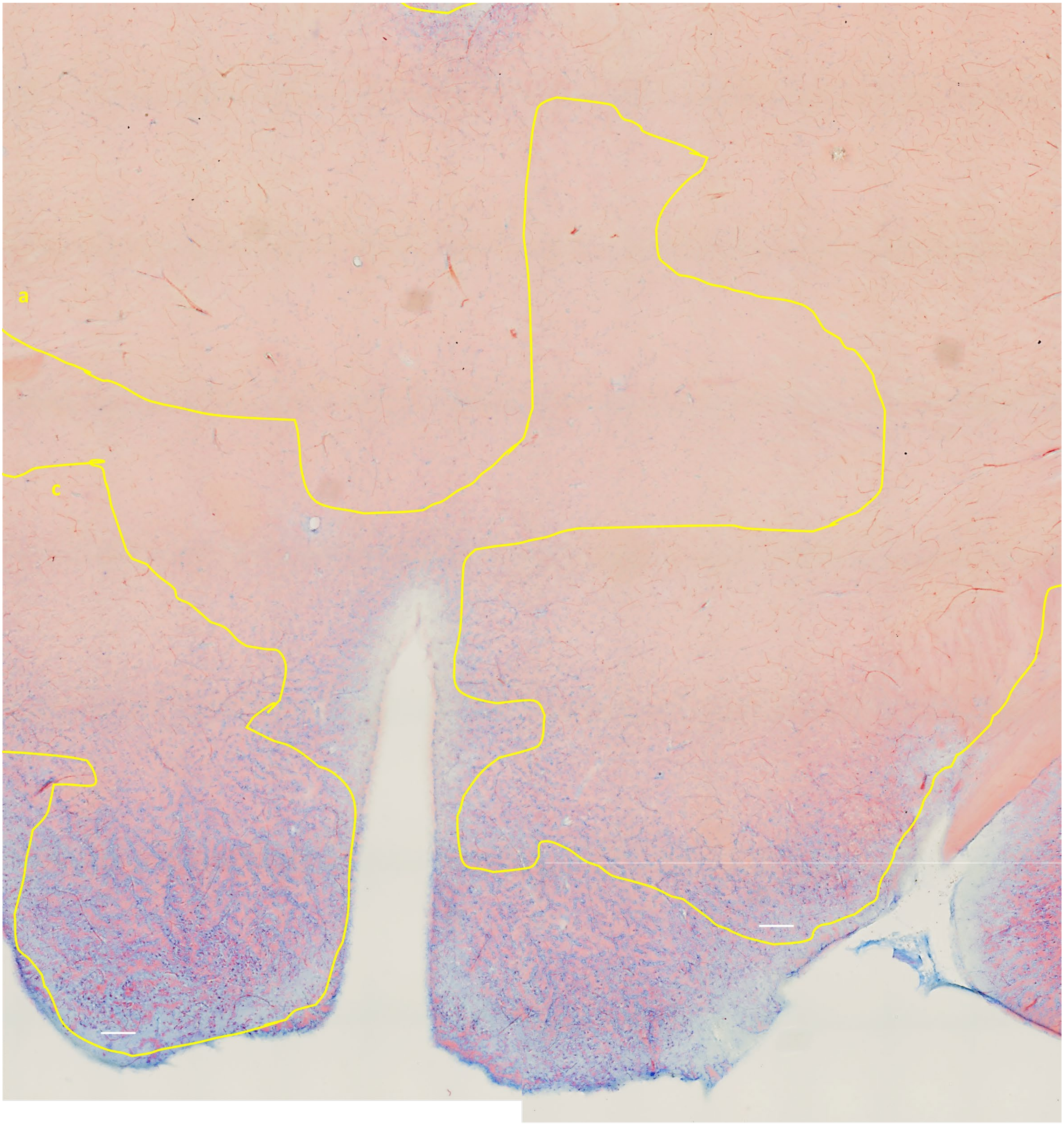

Supplementary Fig. 15. Zoomed in MSB-stained SAH brain clusters a and c in Supplementary Fig. 11. Clusters are outlined in yellow. Images are sharpened for better visualization of microthrombi. Bar = 100μm.

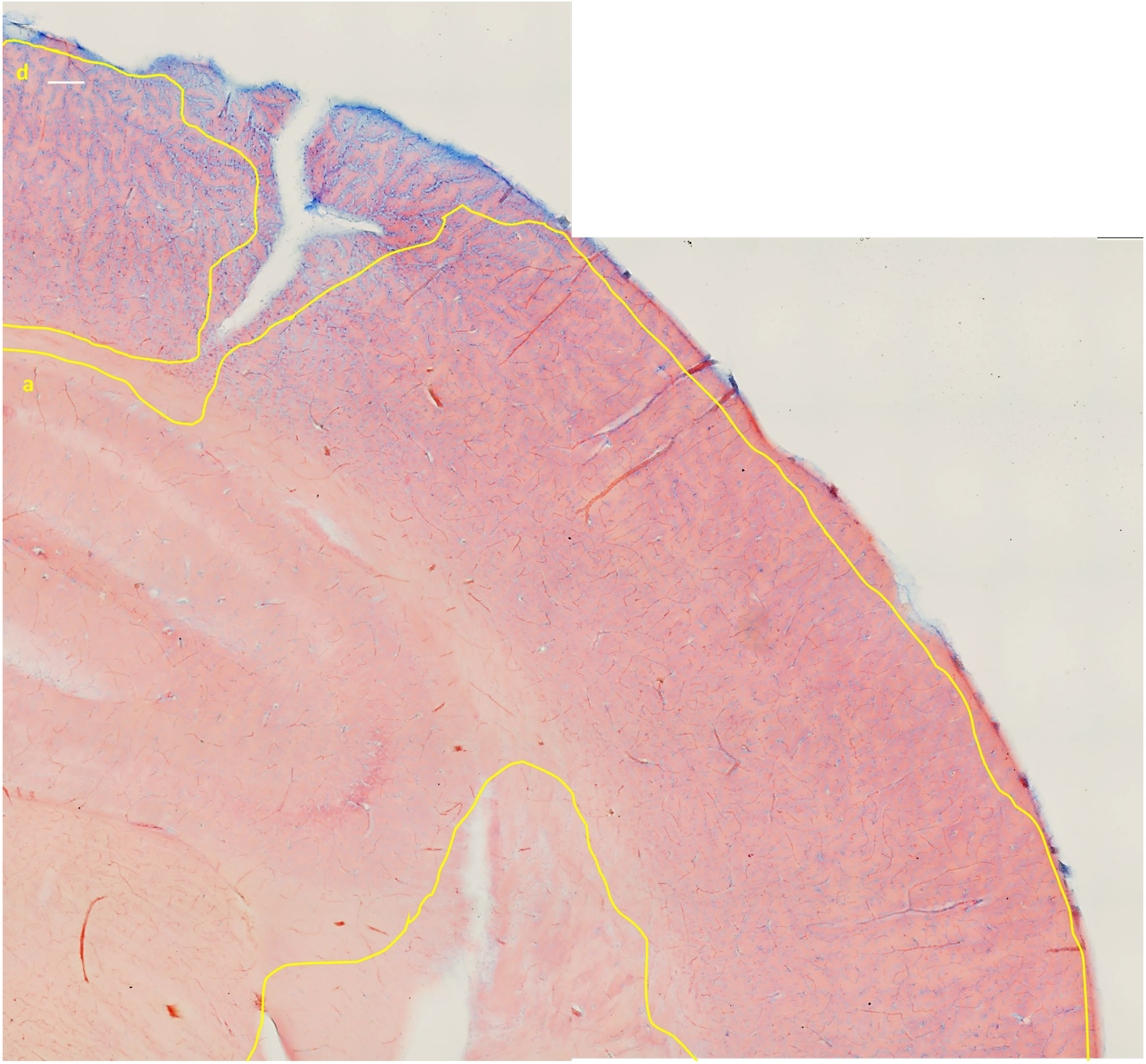

Supplementary Fig. 16. Zoomed in MSB-stained SAH brain clusters a and d in Supplementary Fig. 11. Clusters are outlined in yellow. Images are sharpened for better visualization of microthrombi. Bar = 100μm.

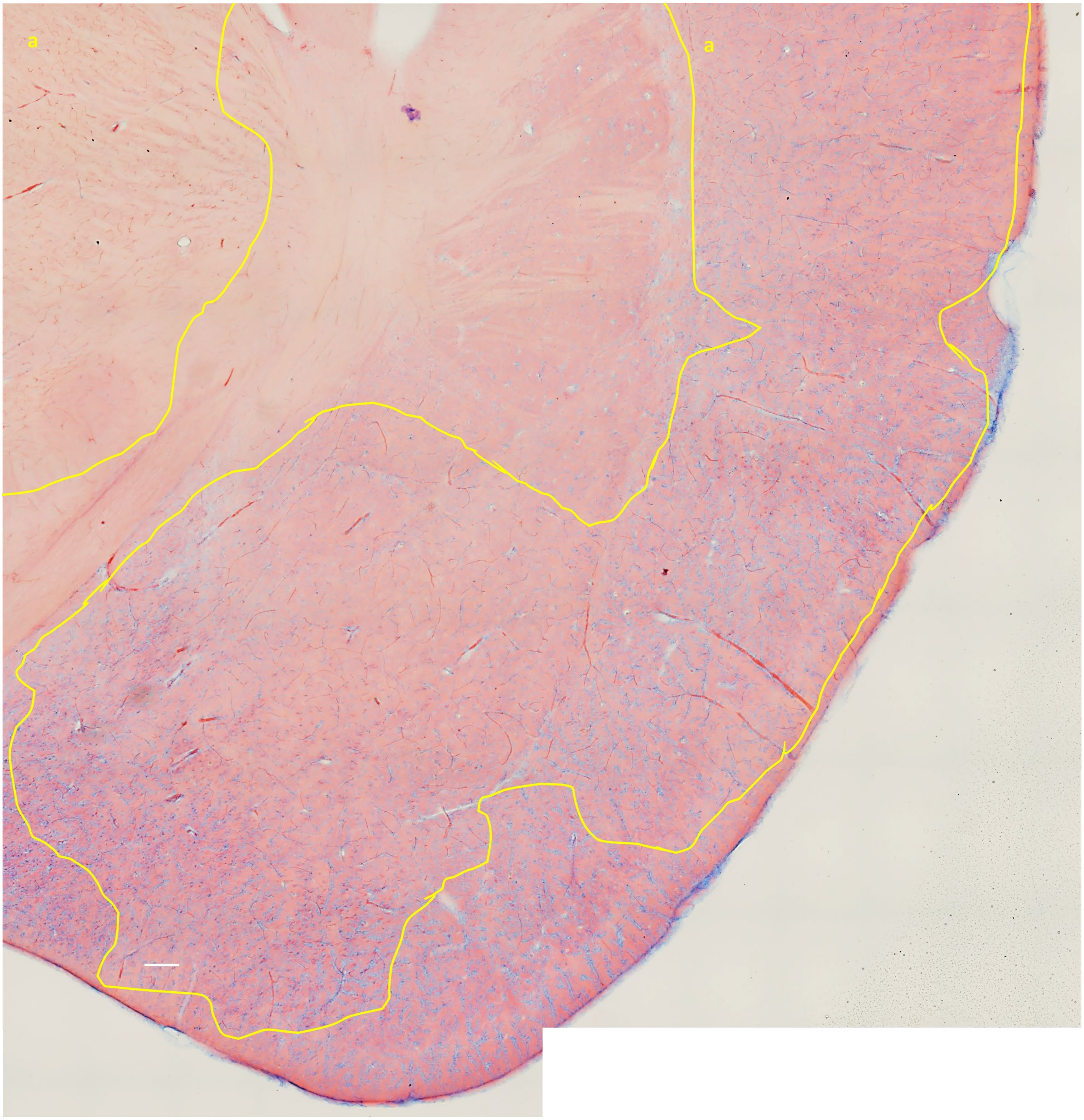

Supplementary Fig. 17. Zoomed in MSB-stained SAH brain cluster a in Supplementary Fig. 11. Clusters are outlined in yellow. Images are sharpened for better visualization of microthrombi. Bar = 100μm.

Supplementary Table 4. Brain Microthrombi Counts and Clusters at -2 from Bregma.

			Microthrombi Localization: Number, Num. of Clusters, % area of brain region					Microthrombi Count
		Ms ID	Cortex	Hippocampus	Thalamus	Hypothalamus	Basal Ganglia	
Day 2	SAH+saline	1	69, 0, 0%	9, 0, 0%	209, 0, 0%	111, 0, 0%	7, 0, 0%	405
		2	424, 3, 2.2%	123, 1, 2.9%	190, 0, 0%	36, 0 0%	2, 0, 0%	775
		3	732, 15, 7.7%	357, 3, 13.6%	466, 8, 6.5%	86, 1, 6.0%	11, 0, 0%	1652
		4	971, 7, 9.1%	156, 4, 9.5%	210, 1, 1.0%	46, 0, 0%	12, 0, 0%	1375
		5	441, 3, 6.0%	5, 0, 0%	50, 0, 0%	12, 0, 0%	4, 0, 0%	512
		6	63, 0, 0%	41, 0, 0%	193, 2, 1.2%	96, 0, 0%	0, 0, 0%	393
		7	519, 3, 1.1%	164, 2, 3.0%	367, 4, 9.1%	207, 2, 11.9%	8, 0, 0%	1267
		8	1206, 7, 6.5%	465, 5, 8.1%	290, 9, 5.9%	390, 5, 8.8%	15, 0, 0%	2366
		9	467, 5, 2.7%	142, 2, 0.8%	477, 8, 8.2%	12, 0, 0%	0, 0, 0%	1098
		10	1039, 2, 7.0%	380, 4, 25.1%	438, 4, 12.7%	134, 2, 4.4%	5, 0, 0%	1996
		11	1794, 7, 10.5%	617, 3, 12.8%	686, 2, 10.7%	321, 0, 0%	20, 0, 0%	3438
	SAH+25% EtOH	1	1058, 4, 3.3%	390, 5, 6.7%	323, 2, 8.2%	158, 0, 0%	10, 0, 0%	1940
		2	487, 4, 1.2%	103, 0, 0%	164, 5, 5.6%	12, 0, 0%	3, 0, 0%	769
		3	1047, 4, 19.4%	16, 0, 0%	79, 0, 0%	42, 0, 0%	7, 0, 0%	1191
		4	561, 3, 1.2%	182, 0, 0%	235, 1, 0.8%	148, 1, 1.3%	0, 0, 0%	1126
		5	578, 1, 0.3%	274, 0, 0%	297, 2, 0.9%	141, 0, 0%	7, 0, 0%	1297
		6	1534, 8, 7.2%	372, 1, 6.0%	278, 5, 3.1%	103, 1, 2.2%	8, 0, 0%	2295
	WT SAH+DT	1	373	28	332	1	0	993
		2	427, 3, 2.7%	217, 2, 3.7%	347, 1, 8.2%	37, 0, 0%	0, 0, 0%	1028
		3	339, 1, 0.5%	156, 1, 6.2%	17, 0, 0%	12, 0, 0%	0, 0, 0%	524
		4	427, 2, 1.4%	2, 0, 0%	66, 0, 0%	4, 0, 0%	0, 0, 0%	499
		5	193, 0, 0%	43, 0, 0%	123, 0, 0%	25, 0, 0%	0, 0, 0%	384
		6	1021, 5, 12.0%	225, 3, 10.6%	243, 2, 4.4%	251, 3, 12.1%	12, 0, 0%	1752
		7	174, 0, 0%	176, 1, 1.0%	166, 0, 0%	25, 0, 0%	0, 0, 0%	541
		8	266, 3, 1.1%	112, 2, 2.9%	59, 0, 0%	11, 0, 0%	2, 0, 0%	450
Day 7	SAH+saline	1	431, 2, 0.7%	82, 0, 0%	174, 0, 0%	39, 0, 0%	5, 0, 0%	734
		2	1279, 7, 7.8%	192, 2, 7.9%	395, 9, 6.6%	87, 0, 0%	12, 0, 0%	1965
		3	207, 0, 0%	21, 0, 0%	109, 0, 0%	7, 0, 0%	0, 0, 0%	344
		4	511, 4, 6.7%	16, 0 , 0%	297, 3, 11.8%	15, 0, 0%	0, 0, 0%	839
		5	267, 0, 0%	43, 0, 0%	249, 1, 0.5%	4, 0, 0%	0, 0, 0%	563
	SAH+25% EtOH	1	502, 0, 0%	67, 0, 0%	218, 1, 0.6%	25, 0, 0%	0, 0, 0%	712
		2	124, 0, 0%	78, 0, 0%	83, 0, 0%	4, 0, 0%	0, 0, 0%	289
		3	2540, 12, 32%	50, 0, 0%	321, 2, 5.9%	251, 2, 5.4%	12, 0, 0%	3174
		4	328, 2, 0.8%	58, 0, 0%	158, 0, 0%	41, 0, 0%	11, 0, 0%	596
		5	340, 0, 0%	88, 0, 0%	86, 1, 6.1%	44, 0, 0%	2, 0, 0%	560
	WT SAH+DT	1	185, 0, 0%	8, 0, 0%	92, 0, 0%	25, 0, 0%	0, 0, 0%	310
		2	322, 2, 1.5%	167, 3, 16.8%	183, 3, 7.2%	35, 0, 0%	0, 0, 0%	707
		3	652, 5, 7.7%	215, 2, 4.0%	169, 3, 7%	303, 4, 11%	12, 0, 0%	1351
		4	212, 2, 1.2%	87, 0, 0%	174, 0, 0%	32, 0, 0%	0, 0, 0%	505
		5	732, 2, 5.2%	60, 0, 0%	48, 0, 0%	32, 0, 0%	0, 0, 0%	872
		6	529, 4, 0.6%	76, 0, 0%	162, 2, 1.0%	0, 0, 0%	0, 0, 0%	767
		7	1552, 3, 20.0%	638, 4, 46.6%	600, 3, 60.8%	433, 2, 2.7%	0, 0, 0%	3223

Supplementary Table 5. Neuroscore, DND, and Ischemia for Mice in Supplementary Table 4.

Mouse	D1 Neuroscore	DND?	Ischemia (% of brain)
SAH+saline 1, day 2	12	N/A	0.8%
SAH+saline 2, day 2	18	N/A	0%
SAH+saline 3, day 2	16	N/A	0%
SAH+saline 4, day 2	18	N/A	0%
SAH+saline 5, day 2	17	N/A	0%
SAH+saline 6, day 2	15	N/A	0.2%
SAH+saline 7, day 2	17	N/A	0%
SAH+saline 8, day 2	17	N/A	3.6%
SAH+saline 9, day 2	16	N/A	0%
SAH+saline 10, day 2	21	N/A	0%
SAH+saline 11, day 2	18	N/A	0%
SAH+saline 12, day 2	15	N/A	0%
SAH+saline 1, day 7	13	D7	7.5%
SAH+saline 2, day 7	15	D6	0%
SAH+saline 3, day 7	13	D4	9.05%
SAH+saline 4, day 7	17	No	0%
SAH+saline 5, day 7	19	D7	12.9%
SAH+25% EtOH 1, day 2	19	N/A	0%
SAH+25% EtOH 2, day 2	14	N/A	0%
SAH+25% EtOH 3, day 2	12	N/A	0%
SAH+25% EtOH 4, day 2	18	N/A	0%
SAH+25% EtOH 5, day 2	16	N/A	0%
SAH+25% EtOH 6, day 2	15	N/A	0%
SAH+25% EtOH 1, day 7	16	No	0%
SAH+25% EtOH 2, day 7	18	No	0%
SAH+25% EtOH 3, day 7	15	D4	3.9%
SAH+25% EtOH 4, day 7	12	No	0%
SAH+25% EtOH 5, day 7	18	No	6.9%
WT SAH+DT 1, day 2	14	N/A	0%
WT SAH+DT 2, day 2	17	N/A	1.7%
WT SAH+DT 3, day 2	17	N/A	4.0%
WT SAH+DT 4, day 2	19	N/A	2.2%
WT SAH+DT 5, day 2	17	N/A	5.2%
WT SAH+DT 6, day 2	17	N/A	34.3%
WT SAH+DT 7, day 2	14	N/A	0%
WT SAH+DT 8, day 2	15	N/A	0%
WT SAH+DT 1, day 7	16	D3	6.8%
WT SAH+DT 2, day 7	14	No	0%
WT SAH+DT 3, day 7	17	No	0%
WT SAH+DT 4, day 7	18	No	3.6%
WT SAH+DT 5, day 7	12	No	0%
WT SAH+DT 6, day 7	18	No	1.9%
WT SAH+DT 7, day 7	13	D7	13.3%

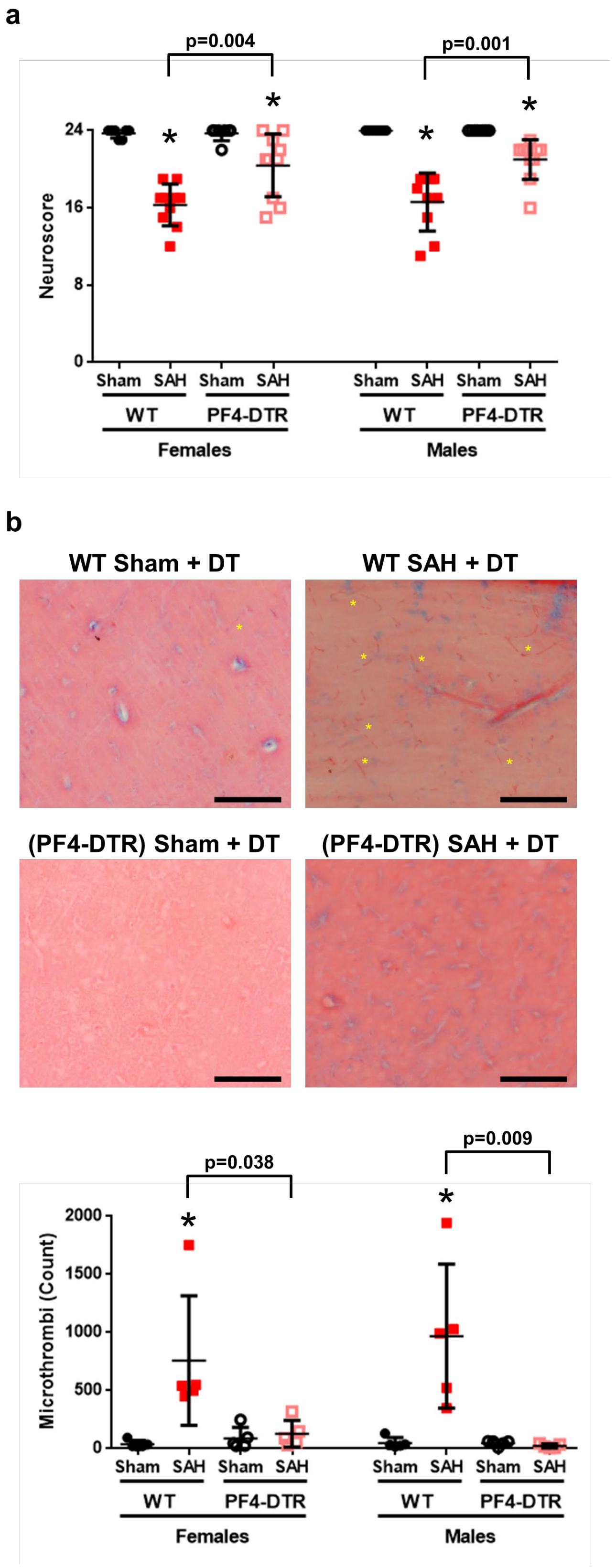

Supplementary Fig. 18. Platelet Depletion Improves Outcomes 2 Days after SAH. After SAH, mice depleted of platelets (PF4-DTR) had better Neuroscores (**a**, n=7 sham/sex/strain, n=10 SAH/sex/strain) and less brain microthrombi (**b**, n=5/sex/group/strain) than non-depleted mice (WT). All mice received the entire DT regimen. Representative images of MSB-stained brain slices showing microthrombi (selected microthrombi marked with yellow *) are best observed when zooming in. Bar=100μm. * $p<0.05$ vs strain-matched Sham.

Supplementary Table 6. Correlational Analysis. The top table examines correlations for Neuroscore on day 1 with day 2 microthrombi count, day 2 cluster count, and day 2 cluster area. The middle table examines correlations among microthrombi count (day 7), cluster count (day 7), cluster area (day 7), infarct area (day 7), Neuroscore on day 1, and DND incidence. The bottom table examines correlations for microthrombi count with clusters (number and area). Total microthrombi and regional microthrombi (cortex, hippocampus, thalamus, hypothalamus) are analyzed for correlation with the clusters (number and area) within the same region.

Day 2

	MT (D2)	Cluster # (D2)	Cluster Area (D2)
NS (D1)	r=0.122, p=0.281	r=0.222, p=0.148	r=0.050, p=0.408

Day 7

	MT (D7)	# clusters MT (D7)	Cluster Area MT (D7)	DND Incidence (D3-D7)	Infarct Area MT (D7)
NS (D1)	r=-0.338, p=0.168	r=-0.205, p=0.363	r=-0.357, p=0.131	r=-0.170, p=0.244	r=0.066, p=0.799
Infarct Area MT (D7)	r=-0.123, p=0.492	r=-0.253, p=0.190	r=-0.218, p=0.244	r=0.559, p=0.026	
DND Incidence MT (D3-D7)	r=0.480, p=0.002	r=0.182, p=0.490	r=0.440, p=0.015		

Data for Day 2 and Day 7 combined

	MT (D2 or D7)				
	Total	Cortex	Hippocampus	Thalamus	Hypothalamus
Cluster Number (D2 or D7)	r=0.863, p<0.001	r=0.,791 p<0.001	r=0.807, p<0.001	r=0.729, p<0.001	r=0.665, p<0.001
Cluster Area (D2 or D7)	r=0.819, p<0.001	r=0.837, p<0.001	r=0.814, p<0.001	r=0.776, p<0.001	r=0.651, p<0.001

Supplementary Table 7. Patient Demographics. Seventeen patients were included into the platelet spreading experiment.

Age	Mean (SD)	58.6 (12.1)
	Range	37-77
Gender	Male	5
	Female	11
Race	American Indian or Alaskan Native	0
	Black or African-American	3
	White	5
	Hispanic or Latino	6
	Asian	1
	Other	1
Hunt Hess on Arrival	0	0
	1	0
	2	4
	3	8
	4	4
	5	0

Supplementary Table 8. Patient Samples and Time Points. Seventeen patients were included into the platelet spreading experiment.

Patient ID	Early Time Point		Delayed Time Point		
	**Day 1**	**Day 2**	**Day 4**	**Day 7**	**Day 10**
1098	Saline, Tirofiban		Saline, Tirofiban	Saline, Tirofiban	Saline, Tirofiban
1018				Saline, Tirofiban	Saline, Tirofiban
1031			Saline, Tirofiban		
1103			Saline, Tirofiban		
1105		Saline, Tirofiban	Saline, Tirofiban		
A0	Saline, Tirofiban				
A1	Saline, Tirofiban				
A2	Saline, Tirofiban				
1066	Saline, Tirofiban				
0707				Saline, Tirofiban	
0711			Saline, Tirofiban	Saline, Tirofiban	
0712		Saline, A3P5PS	Saline, A3P5PS	Saline, A3P5PS	Saline, A3P5PS
0713	Saline, A3P5PS		Saline, A3P5PS	Saline, A3P5PS	Saline, A3P5PS
0714				Saline, A3P5PS	Saline, A3P5PS
0715		Saline, A3P5PS	Saline, A3P5PS		Saline, A3P5PS
0719		Saline, A3P5PS			
0708		Saline, Tirofiban			

Statistical Reports

Supplementary Table 9. Statistical Report for Plasma Markers of Platelet Activators Over Time (Fig. 2). PAF: Kruskal-Wallis test statistic = 19.011, DoF=4, p<0.001. TXB_2: Kruskal-Wallis test statistic = 12.228, DoF=4, p=0.016. Thrombin: One-way ANOVA, F=2.177, DoF=4, p=0.102. PF4: One-way ANOVA with Tukey post-hoc, F=1.241, DoF=4, p=0.319. Statistically significant comparisons are highlighted in bold.

	PAF		TXB_2		Thrombin	PF4
	Test Statistic	p-Value	Test Statistic	p-Value	Mean Difference	Mean Difference
Sham vs SAH Day 1	15.500	**0.002**	14.167	**0.005**	2.53	39.6
Sham vs SAH Day 3	1.333	0.262	9.333	0.066	4.43	55.6
Sham vs SAH Day 5	14.000	**0.006**	12.167	**0.017**	1.30	16.2
Sham vs SAH Day 7	4.333	0.394	1.833	0.718	1.47	92.5
SAH Day 1 vs SAH Day 3	16.833	**<0.001**	4.833	0.342	1.90	95.3
SAH Day 1 vs SAH Day 5	1.500	0.295	2.000	0.694	1.23	23.5
SAH Day 1 vs SAH Day 7	11.167	**0.028**	12.333	**0.015**	1.06	132
SAH Day 3 vs SAH Day 5	15.333	**0.003**	2.833	0.577	3.13	71.8
SAH Day 3 vs SAH Day 7	5.667	0.265	7.500	0.140	2.96	36.9
SAH Day 5 vs SAH Day 7	9.667	0.057	10.333	**0.042**	0.173	108

Supplementary Table 10. Statistical Report for Exogenous Activation of Platelets with PAF Neuroscore (Fig. 3b and 3d). 2-Day Study: Kruskall-Wallis test with Bonferroni post-hoc. 2-Day Study Female – Test statistic=13.560, DoF=2, p=0.001. 2-Day Study Male – Test statistic=14.526, DoF=2, p<0.001. 7-Day Study: Day 1 NS (Neuroscore performed 1-day post-SAH): One-way ANOVA with Bonferroni post-hoc; F=27.565, DoF=2, p<0.001. Day 4 NS (Neuroscore performed 1-day post-PAF injection): Kruskall-Wallis test with Bonferroni post-hoc; Test statistic=10.872, DoF=2, p=0.004. Statistically significant comparisons are highlighted in bold.

			Test Statistic	p-Value
2-Day Study	Female	Sham+PAF vs SAH+Saline	14.73	**0.001**
		Sham+PAF vs SAH+PAF	14.27	**0.001**
		SAH+Saline vs SAH+PAF	0.455	1.000
	Male	Sham+PAF vs SAH+Saline	16.167	**0.001**
		Sham+PAF vs SAH+PAF	17.167	**0.002**
		SAH+Saline vs SAH+PAF	1.000	1.000
7-Day Study	Day 1 NS	Sham+PAF vs SAH+Saline	N/A	**<0.001**
		Sham+PAF vs SAH+PAF	N/A	**<0.001**
		SAH+Saline vs SAH+PAF	N/A	0.226
	Day 4 NS	Sham+PAF vs SAH+Saline	1.518	0.387
		Sham+PAF vs SAH+PAF	3.157	**0.005**
		SAH+Saline vs SAH+PAF	2.290	0.066

Supplementary Table 11. Statistical Report for Exogenous Activation of Platelets with PAF Neuroscore for the 7-Day Study (Fig. 3d). Friedman test (Chi-square=82.483, Dof=6, p<0.001) followed by Kruskall-Wallis test with Bonferroni post-hoc for each day. Day 1: Test Statistic=24.695, DoF=2, p<0.001. Day 2: Test Statistic=13.334, DoF=2, p=0.001. Day 3: Test Statistic=7.658, DoF=2, p=0.022. Day 4: Test Statistic=12.335, DoF=2, p=0.002. Day 5: Test Statistic=13.268, DoF=2, p=0.001. Day 6: Test Statistic=5.459, DoF=2, p=0.065. Day 7: Test Statistic=3.580, DoF=2, p=0.167.

		Test Statistic	p-Value
Day 1	Sham+PAF vs SAH+Saline	34.808	**<0.001**
	Sham+PAF vs SAH+PAF	25.192	**0.001**
	SAH+Saline vs SAH+PAF	9.615	0.137
Day 2	Sham+PAF vs SAH+Saline	21.630	**0.005**
	Sham+PAF vs SAH+PAF	24.957	**0.001**
	SAH+Saline vs SAH+PAF	3.327	1.000
Day 3	Sham+PAF vs SAH+Saline	8.971	0.538
	Sham+PAF vs SAH+PAF	17.279	**0.029**
	SAH+Saline vs SAH+PAF	8.308	0.210
Day 4	Sham+PAF vs SAH+Saline	10.375	0.177
	Sham+PAF vs SAH+PAF	19.688	**0.002**
	SAH+Saline vs SAH+PAF	9.313	0.072
Day 5	Sham+PAF vs SAH+Saline	9.455	0.228
	Sham+PAF vs SAH+PAF	19.344	**0.002**
	SAH+Saline vs SAH+PAF	9.889	**0.043**

Supplementary Table 12. Statistical Report for Microthrombi Count after Exogenous Activation of Platelets with PAF (Fig. 3c and f). 2-Day study: Female data was analyzed with a One-way ANOVA with Bonferroni post-hoc (F=28.466, p<0.001), male data was analyzed using a Kruskal-Wallis test (H=11.180, p=0.004). 7-Day study: One-way ANOVA with Bonferroni post-hoc (F=15.444, p<0.001).

			p-Value
2-Day Study	Female	Sham+PAF vs SAH+Saline	**0.012**
		Sham+PAF vs SAH+PAF	**<0.001**
		SAH+Saline vs SAH+PAF	**0.005**
	Male	Sham+PAF vs SAH+Saline	**0.048**
		Sham+PAF vs SAH+PAF	**<0.001**
		SAH+Saline vs SAH+PAF	0.179
7-Day Study	Female	Sham+PAF vs SAH+Saline	0.167
		Sham+PAF vs SAH+PAF	**<0.001**
		SAH+Saline vs SAH+PAF	**0.011**

Supplementary Table 13. Statistical Report of Neuroscore for LIGHT$^{-/-}$ Mice (Platelet Hyperactivity) (Fig. 4a). WT: C57BL/6J mice. Friedman test followed by Kruskall-Wallis test with Bonferroni post-hoc for each day. Male (Chi-square=100.518, Dof=6, p<0.001): Day 1: Test Statistic=20.042, DoF=2, p<0.001. Day 2: Test Statistic=16.957, DoF=2, p<0.001. Day 3: Test Statistic=17.752, DoF=2, p<0.001. Day 4: Test Statistic=6.068, DoF=2, p=0.048. Day 5: Test Statistic=4.800, DoF=2, p=0.091. Day 6: Test Statistic=6.531, DoF=2, p=0.038. Day 7: Test Statistic=2.489, DoF=2, p=0.288. Female (Chi-square=69.065, Dof=6, p<0.001): Day 1: Test Statistic=22.285, DoF=2, p<0.001. Day 2: Test Statistic=7.033, DoF=2, p=0.030. Day 3: Test Statistic=8.167, DoF=2, p=0.017. Day 4: Test Statistic=8.543, DoF=2, p=0.014. Day 5: Test Statistic=8.780, DoF=2, p=0.012. Day 6: Test Statistic=4.157, DoF=2, p=0.125. Day 7: Test Statistic=3.061, DoF=2, p=0.216.

			Test Statistic	p-Value
Female	Day 1	LIGHT$^{-/-}$ Sham vs LIGHT$^{-/-}$ SAH	25.905	**0.002**
		LIGHT$^{-/-}$ Sham vs WT SAH	36.051	**<0.001**
		LIGHT$^{-/-}$ SAH vs WT SAH	10.147	0.111
	Day 2	LIGHT$^{-/-}$ Sham vs LIGHT$^{-/-}$ SAH	19.360	**0.024**
		LIGHT$^{-/-}$ Sham vs WT SAH	16.409	0.082
		LIGHT$^{-/-}$ SAH vs WT SAH	2.951	1.000
	Day 3	LIGHT$^{-/-}$ Sham vs LIGHT$^{-/-}$ SAH	18.946	**0.018**
		LIGHT$^{-/-}$ Sham vs WT SAH	18.186	**0.025**
		LIGHT$^{-/-}$ SAH vs WT SAH	0.76	1.000
	Day 4	LIGHT$^{-/-}$ Sham vs LIGHT$^{-/-}$ SAH	18.913	**0.012**
		LIGHT$^{-/-}$ Sham vs WT SAH	16.700	**0.031**
		LIGHT$^{-/-}$ SAH vs WT SAH	2.213	1.000
	Day 5	LIGHT$^{-/-}$ Sham vs LIGHT$^{-/-}$ SAH	16.593	**0.015**
		LIGHT$^{-/-}$ Sham vs WT SAH	8.620	0.420
		LIGHT$^{-/-}$ SAH vs WT SAH	7.973	0.166
Male	Day 1	LIGHT$^{-/-}$ Sham vs LIGHT$^{-/-}$ SAH	25.850	**0.002**
		LIGHT$^{-/-}$ Sham vs WT SAH	33.803	**<0.001**
		LIGHT$^{-/-}$ SAH vs WT SAH	7.953	0.297
	Day 2	LIGHT$^{-/-}$ Sham vs LIGHT$^{-/-}$ SAH	12.601	0.247
		LIGHT$^{-/-}$ Sham vs WT SAH	27.095	**0.001**
		LIGHT$^{-/-}$ SAH vs WT SAH	14.494	**0.007**
	Day 3	LIGHT$^{-/-}$ Sham vs LIGHT$^{-/-}$ SAH	14.607	0.102
		LIGHT$^{-/-}$ Sham vs WT SAH	27.609	**<0.001**
		LIGHT$^{-/-}$ SAH vs WT SAH	13.002	**0.014**
	Day 4	LIGHT$^{-/-}$ Sham vs LIGHT$^{-/-}$ SAH	12.768	0.197
		LIGHT$^{-/-}$ Sham vs WT SAH	17.419	**0.042**
		LIGHT$^{-/-}$ SAH vs WT SAH	4.651	0.941
	Day 6	LIGHT$^{-/-}$ Sham vs LIGHT$^{-/-}$ SAH	13.286	0.109
		LIGHT$^{-/-}$ Sham vs WT SAH	16.308	**0.033**
		LIGHT$^{-/-}$ SAH vs WT SAH	3.023	1.000

Supplementary Table 14. Statistical Report for Platelet Depletion on Day 1 Neuroscore in the 2-Day Study (Supplementary Fig. 18a). t-Test or Mann–Whitney U test as appropriate. All mice received injections of diphtheria toxin on days -5, -3, and -1. WT: C57BL/6J mice.

		t or U	p-Value
Female	WT Sham vs WT SAH	8.342	**<0.001**
	WT Sham vs PF4-DTR Sham	0	1.000
	PF4-DTR Sham vs PF4-DTR SAH	3.087	**0.011**
	WT SAH vs PF4-DTR SAH	2.286	**0.035**
Male	WT Sham vs WT SAH	6.290	**<0.001**
	WT Sham vs PF4-DTR Sham	N/A	1.000
	PF4-DTR Sham vs PF4-DTR SAH	4.737	**<0.001**
	WT SAH vs PF4-DTR SAH	2.849	**0.01**

Supplementary Table 15. Statistical Report of Day 2 Microthrombi Count for Platelet Depletion (Supplementary Fig. 18b). t-tests (Mann–Whitney U tests for WT sham vs WT SAH). All mice received injections of diphtheria toxin on days -5, -3, and -1. WT: C57BL/6J mice.

		Test Statistic	p-Value
Female	WT Sham vs WT SAH	2.884	**0.044**
	WT Sham vs PF4-DTR Sham	1.113	0.298
	PF4-DTR Sham vs PF4-DTR SAH	0.596	0.568
	WT SAH vs PF4-DTR SAH	2.479	**0.038**
Male	WT Sham vs WT SAH	3.302	**0.029**
	WT Sham vs PF4-DTR Sham	0.000	1.000
	PF4-DTR Sham vs PF4-DTR SAH	1.996	0.081
	WT SAH vs PF4-DTR SAH	3.405	**0.009**

Supplementary Table 16. Statistical Report for Platelet Depletion on Longitudinal Neuroscore in Female Mice for the 7-Day Study (Fig. 5a). Friedman test followed by t-test or Mann–Whitney U test (as appropriate) for each day. WT Sham vs WT SAH: Chi-Square=45.166, DoF=6, p<0.001. PF4-DTR Sham vs PF4-DTR SAH: Chi-Square=35.466, DoF=6, p<0.001. WT SAH vs PF4-DTR SAH: Chi-Square=89.095, DoF=6, p<0.001. p-values for the pairwise testing is compared to the Bonferroni adjusted p-value for multiple comparisons; all p-values less than p<0.017. are considered to be statistically significant. All mice received injections of diphtheria toxin on days -5, -3, and -1. WT: C57BL/6J mice.

		Test Statistic	p-Value
Day 1	WT Sham vs WT SAH	13.760	**<0.001**
	PF4-DTR Sham vs PF4-DTR SAH	7.410	**<0.001**
	WT SAH vs PF4-DTR SAH	5.522	**<0.001**
Day 2	WT Sham vs WT SAH	4.765	**<0.001**
	PF4-DTR Sham vs PF4-DTR SAH	4.104	**<0.001**
	WT SAH vs PF4-DTR SAH	1.513	0.136
Day 3	WT Sham vs WT SAH	3.035	**0.005**
	PF4-DTR Sham vs PF4-DTR SAH	3.949	**<0.001**
	WT SAH vs PF4-DTR SAH	0.223	0.824
Day 4	WT Sham vs WT SAH	2.294	0.029
	PF4-DTR Sham vs PF4-DTR SAH	1.327	0.193
	WT SAH vs PF4-DTR SAH	0.927	0.358
Day 5	WT Sham vs WT SAH	1.009	0.321
	PF4-DTR Sham vs PF4-DTR SAH	1.338	0.190
	WT SAH vs PF4-DTR SAH	0.671	0.505
Day 6	WT Sham vs WT SAH	1.876	0.073
	PF4-DTR Sham vs PF4-DTR SAH	0.860	0.396
	WT SAH vs PF4-DTR SAH	0.355	0.724
Day 7	WT Sham vs WT SAH	2.023	0.056
	PF4-DTR Sham vs PF4-DTR SAH	1.071	0.292
	WT SAH vs PF4-DTR SAH	0.639	0.526

Supplementary Table 17. Statistical Report for Platelet Depletion on Longitudinal Neuroscore in Male Mice (Fig. 5a). Friedman test followed by t-test or Mann–Whitney U test (as appropriate) for each day. WT Sham vs WT SAH: Chi-Square=67.560, DoF=6, p<0.001. PF4-DTR Sham vs PF4-DTR SAH: Chi-Square=46.381, DoF=6, p<0.001. WT SAH vs PF4-DTR SAH: Chi-Square=102.110, DoF=6, p<0.001. p-values for the pairwise testing is compared to the Bonferroni adjusted p-value for multiple comparisons; all p-values less than p<0.017. are considered to be statistically significant. All mice received injections of diphtheria toxin on days -5, -3, and -1. WT: C57BL/6J mice.

		t or U	p-Value
Day 1	WT Sham vs WT SAH	7.595	**<0.001**
	PF4-DTR Sham vs PF4-DTR SAH	5.731	**<0.001**
	WT SAH vs PF4-DTR SAH	5.761	**<0.001**
Day 2	WT Sham vs WT SAH	2.453	0.083
	PF4-DTR Sham vs PF4-DTR SAH	3.357	0.090
	WT SAH vs PF4-DTR SAH	0.538	0.592
Day 3	WT Sham vs WT SAH	3.464	0.080
	PF4-DTR Sham vs PF4-DTR SAH	2.905	0.113
	WT SAH vs PF4-DTR SAH	0.827	0.412
Day 4	WT Sham vs WT SAH	4.630	**<0.001**
	PF4-DTR Sham vs PF4-DTR SAH	2.373	0.024
	WT SAH vs PF4-DTR SAH	4.101	**<0.001**
Day 5	WT Sham vs WT SAH	2.550	**0.016**
	PF4-DTR Sham vs PF4-DTR SAH	1.269	0.212
	WT SAH vs PF4-DTR SAH	1.216	0.229
Day 6	WT Sham vs WT SAH	2.943	**0.006**
	PF4-DTR Sham vs PF4-DTR SAH	2.382	0.023
	WT SAH vs PF4-DTR SAH	1.202	0.235
Day 7	WT Sham vs WT SAH	3.035	**0.006**
	PF4-DTR Sham vs PF4-DTR SAH	1.027	0.311
	WT SAH vs PF4-DTR SAH	1.485	0.144

Supplementary Table 18. Statistical Report of Day 7 Microthrombi Count for Platelet Depletion (Fig. 5c). t-tests (Mann–Whitney U test for female WT sham vs WT SAH). All mice received injections of diphtheria toxin on days -5, -3, and -1. WT: C57BL/6J mice.

		Test Statistic	p-Value
Female	WT Sham vs WT SAH	7.225	**<0.001**
	WT Sham vs PF4-DTR Sham	0.472	0.647
	PF4-DTR Sham vs PF4-DTR SAH	1.459	0.175
	WT SAH vs PF4-DTR SAH	6.849	**<0.001**
Male	WT Sham vs WT SAH	3.632	**0.014**
	WT Sham vs PF4-DTR Sham	0.184	0.857
	PF4-DTR Sham vs PF4-DTR SAH	2.455	**0.034**
	WT SAH vs PF4-DTR SAH	2.354	**0.040**

Supplementary Table 19. Statistical Report for Platelet Receptor Antagonism on Day 1 Neuroscore for the 2-Day Study (Fig. 6a). Kruskall-Wallis with Bonferroni post-hoc. Female mice SAH+Vehicle 1 vs SAH+Vehicle 2: t=1.023, p=0.328. Male mice SAH+Vehicle 1 vs SAH+Vehicle 2: t=1.386, p=0.177.

		Test Statistic	p-Value
Female	Sham vs SAH+Vehicle 1	44.028	**<0.001**
	Sham vs SAH+(AP5PS+Clopidogrel)	30.000	**0.001**
	Sham vs SAH+Daltroban	11.800	1.000
	Sham vs SAH+ML354	27.306	**0.006**
	Sham vs SAH+WEB2086	17.250	0.321
	Sham vs SAH+Vehicle 2	17.000	**<0.001**
	Sham + SAH+Tirofiban	6.567	0.257
	SAH+Vehicle 1 vs SAH+(AP5PS+Clopidogrel)	14.028	1.000
	SAH+Vehicle 1 vs SAH+Daltroban	32.228	**<0.001**
	SAH+Vehicle 1 vs SAH+ML354	16.722	0.515
	SAH+Vehicle 1 vs SAH+WEB2086	26.778	**0.008**
	SAH+Vehicle 2 vs SAH+Tirofiban	10.433	**0.019**
	SAH+(AP5PS+Clopidogrel) vs SAH+Daltroban	18.200	0.228
	SAH+(AP5PS+Clopidogrel) vs SAH+ML354	2.694	1.000
	SAH+(AP5PS+Clopidogrel) vs SAH+WEB2086	12.750	1.000
	SAH+Daltroban vs SAH+ML354	15.506	0.662
	SAH+Daltroban vs SAH+WEB2086	5.450	1.000
	SAH+ML354 vs SAH+WEB2086	10.056	1.000
Male	Sham + SAH+Vehicle 2 (Tirofiban comparison)	18.250	**<0.001**
	Sham + SAH+Tirofiban	7.500	0.134
	SAH+Vehicle 2 vs SAH+Tirofiban	10.750	**0.015**

Supplementary Table 20. Statistical Report for Platelet Receptor Antagonism on Day 1 Neuroscore for the 2-Day Study (Fig. 6a). One-way ANOVA with Tukey post-hoc.

		Mean Difference	95% Con. Interval	p-Value
Male	Sham vs SAH+Vehicle 1	8.500	6.23, 10.73	**<0.001**
	Sham vs SAH+(AP5PS+Clopidogrel)	2.100	0.13, 4.33	0.077
	Sham vs SAH+Daltroban	2.400	0.17, 4.43	**0.028**
	Sham vs SAH+ML354	2.200	0.03, 4.43	0.056
	Sham vs SAH+WEB2086	2.200	0.03, 4.43	0.056
	SAH+Vehicle 1 vs SAH+ML354	6.400	4.42, 8.63	**<0.001**
	SAH+Vehicle 1 vs SAH+WEB2086	6.100	3.87, 8.33	**<0.001**
	SAH+Vehicle 1 vs SAH+(AP5PS+Clopidogrel)	6.300	4.07, 8.53	**<0.001**
	SAH+Vehicle 1 vs SAH+Daltroban	6.300	4.07, 8.53	**<0.001**
	SAH+(AP5PS+Clopidogrel) vs SAH+Daltroban	0.300	1.93, 2.53	0.999
	SAH+(AP5PS+Clopidogrel) vs SAH+ML354	0.100	2.13, 2.33	1.000
	SAH+(AP5PS+Clopidogrel) vs SAH+WEB2086	0.100	2.13, 2.33	1.000
	SAH+Daltroban vs SAH+ML354	0.200	2.03, 2.43	1.000
	SAH+Daltroban vs SAH+WEB2086	0.200	2.03, 2.43	1.000
	SAH+ML354 vs SAH+WEB2086	0.000	2.23, 2.23	1.000

Supplementary Table 21. Statistical Report of Day 2 Microthrombi Count for Platelet Receptor Antagonism (Fig. 6b). One-way ANOVA with Bonferroni post-hoc for female aggregation inhibitor analysis (vehicle 2, F=24.928, p<0.001). Kruskall-Wallis test for female activation inhibitor analysis (H=21.049, p<0.001), male activation inhibitor analysis (H=20.517, p<0.001), and male aggregation inhibitor analysis (H=15.158, p<0.001). Female mice SAH+Vehicle 1 vs SAH+Vehicle 2: U=1.241, p=0.257. Male mice SAH+Vehicle 1 vs SAH+Vehicle 2: U=1.482, p=0.191.

		p-Value
Female	Sham vs SAH+Vehicle 1	**<0.001**
	Sham vs SAH+(AP5PS+Clopidogrel)	**<0.001**
	Sham vs SAH+Daltroban	0.203
	Sham vs SAH+ML354	**0.004**
	Sham vs SAH+WEB2086	0.150
	Sham vs SAH+Vehicle 2	**<0.001**
	Sham vs SAH+Tirofiban	0.399
	SAH+Vehicle 1 vs SAH+(AP5PS+Clopidogrel)	0.784
	SAH+Vehicle 1 vs SAH+Daltroban	**0.015**
	SAH+Vehicle 1 vs SAH+ML354	0.403
	SAH+Vehicle 1 vs SAH+WEB2086	**0.023**
	SAH+Vehicle 2 vs SAH+Tirofiban	**<0.001**
	SAH+(AP5PS+Clopidogrel) vs SAH+Daltroban	**0.030**
	SAH+(AP5PS+Clopidogrel) vs SAH+ML354	0.574
	SAH+(AP5PS+Clopidogrel) vs SAH+WEB2086	**0.045**
	SAH+Daltroban vs SAH+ML354	0.109
	SAH+Daltroban vs SAH+WEB2086	0.869
	SAH+ML354 vs SAH+WEB2086	0.150
Male	Sham vs SAH+Vehicle 1	**<0.001**
	Sham vs SAH+(AP5PS+Clopidogrel)	0.092
	Sham vs SAH+Daltroban	0.077
	Sham vs SAH+ML354	0.055
	Sham vs SAH+WEB2086	**0.011**
	Sham vs SAH+Vehicle 2	**<0.001**
	Sham vs SAH+Tirofiban	0.052
	SAH+Vehicle 1 vs SAH+(AP5PS+Clopidogrel)	**0.006**
	SAH+Vehicle 1 vs SAH+Daltroban	**0.008**
	SAH+Vehicle 1 vs SAH+ML354	**0.013**
	SAH+Vehicle 1 vs SAH+WEB2086	0.062
	SAH+Vehicle 2 vs SAH+Tirofiban	0.052
	SAH+(AP5PS+Clopidogrel) vs SAH+Daltroban	0.934
	SAH+(AP5PS+Clopidogrel) vs SAH+ML354	0.816
	SAH+(AP5PS+Clopidogrel) vs SAH+WEB2086	0.388
	SAH+Daltroban vs SAH+ML354	0.880
	SAH+Daltroban vs SAH+WEB2086	0.435
	SAH+ML354 vs SAH+WEB2086	0.529

Supplementary Table 22. Statistical Report for Platelet Activation Receptor Antagonism on Neuroscore for the 7-Day Study (Fig. 7a). Friedman test (Chi-squared=90.096, DoF=6, p<0.001) followed by Kruskal-Wallis with Bonferroni post-hoc for each day. For activation inhibitor analysis (vehicle 1) comparisons – Day 1: F=43.884, DoF=3, p<0.001, Day 2: F=10.482, DoF=3, p=0.015, Day 3: F=11.200, DoF=3, p=0.011, Day 4: F=8.993, DoF=3, p=0.029, Day 5: F=6.145, DoF=3, p=0.105, Day 6: F=12.373, DoF=3, p=0.006, Day 7: F=3.648, DoF=3, p=0.302.

		Test Statistic	p-Value
Day 1	Sham vs SAH+Vehicle 1	49.300	**<0.001**
	Sham vs SAH+Daltroban	23.300	**0.017**
	Sham + SAH+WEB2086	22.600	**0.023**
	SAH+Vehicle 1 vs SAH+Daltroban	26.000	**<0.001**
	SAH+Vehicle 1 vs SAH+WEB2086	26.700	**<0.001**
	SAH+Daltroban vs SAH+WEB2086	0.700	1.000
Day 2	Sham vs SAH+Vehicle 1	23.200	**0.008**
	Sham vs SAH+Daltroban	18.100	0.075
	Sham + SAH+WEB2086	16.450	0.139
	SAH+Vehicle 1 vs SAH+Daltroban	5.100	1.000
	SAH+Vehicle 1 vs SAH+WEB2086	6.750	1.000
	SAH+Daltroban vs SAH+WEB2086	1.650	1.000
Day 3	Sham vs SAH+Vehicle 1	22.275	**0.009**
	Sham vs SAH+Daltroban	14.026	0.288
	Sham + SAH+WEB2086	19.789	**0.032**
	SAH+Vehicle 1 vs SAH+Daltroban	8.249	0.938
	SAH+Vehicle 1 vs SAH+WEB2086	2.486	1.000
	SAH+Daltroban vs SAH+WEB2086	5.763	1.000
Day 4	Sham vs SAH+Vehicle 1	17.171	**0.031**
	Sham vs SAH+Daltroban	5.547	1.000
	Sham + SAH+WEB2086	10.100	0.548
	SAH+Vehicle 1 vs SAH+Daltroban	11.624	0.157
	SAH+Vehicle 1 vs SAH+WEB2086	7.071	1.000
	SAH+Daltroban vs SAH+WEB2086	4.553	1.000
Day 6	Sham vs SAH+Vehicle 1	8.625	0.319
	Sham vs SAH+Daltroban	0.000	1.000
	Sham + SAH+WEB2086	11.179	0.057
	SAH+Vehicle 1 vs SAH+Daltroban	8.625	0.181
	SAH+Vehicle 1 vs SAH+WEB2086	2.554	1.000
	SAH+Daltroban vs SAH+WEB2086	11.179	**0.020**

Supplementary Table 23. Statistical Report for Platelet Aggregation Receptor Antagonism on Neuroscore for the 7-Day Study (Fig. 7a). Friedman test (Chi-squared=37.210, DoF=6, p<0.001) followed by Kruskal-Wallis with Bonferroni post-hoc for each day. For aggregation inhibitor analysis (vehicle 2) comparisons – Day 1: F=30.534, DoF=2, p<0.001. Day 2: F=15.601, DoF=2, p<0.001, Day 3: F=12.951, DoF=2, p=0.002, Day 4: F=6.331, DoF=2, p=0.042, Day 5: F=10.825, DoF=2, p=0.004, Day 6: F=9.131, DoF=2, p=0.010, Day 7: F=1.679, DoF=2, p=0.432.

		Test Statistic	p-Value
Day 1	Sham vs SAH+Vehicle 2	29.750	**0.001**
	Sham vs SAH+Tirofiban	13.750	**0.041**
	SAH+Vehicle 2 vs SAH+Tirofiban	16.000	**0.001**
Day 2	Sham vs SAH+Vehicle 2	21.250	**<0.001**
	Sham vs SAH+Tirofiban	13.750	**0.032**
	SAH+Vehicle 2 vs SAH+Tirofiban	7.500	0.264
Day 3	Sham vs SAH+Vehicle 2	18.342	**0.001**
	Sham vs SAH+Tirofiban	10.750	0.102
	SAH+Vehicle 2 vs SAH+Tirofiban	7.592	0.211
Day 4	Sham vs SAH+Vehicle 2	10.900	**0.045**
	Sham vs SAH+Tirofiban	4.425	0.894
	SAH+Vehicle 2 vs SAH+Tirofiban	6.475	0.252
Day 5	Sham vs SAH+Vehicle 2	13.969	**0.007**
	Sham vs SAH+Tirofiban	4.224	1.000
	SAH+Vehicle 2 vs SAH+Tirofiban	9.745	**0.036**
Day 6	Sham vs SAH+Vehicle 2	12.545	**0.013**
	Sham vs SAH+Tirofiban	9.667	**0.043**
	SAH+Vehicle 2 vs SAH+Tirofiban	2.879	1.000

Supplementary Table 24. Statistical Report of Day 7 Microthrombi Count for Platelet Receptor Antagonism (Fig. 7c). Kruskall-Wallis with Bonferroni post-hoc: activation inhibitor analysis (Vehicle 1, H=18.540, p<0.001), aggregation inhibitor analysis (vehicle 2, H=10.760, p=0.005). SAH+Vehicle 1 vs SAH+Vehicle 2: t=0.361, p=0.725.

	Test Statistic	p-Value
Sham vs SAH+Vehicle 1	15.167	**0.001**
Sham vs SAH+Daltroban	5.167	1.000
Sham vs SAH+WEB2086	13.667	**0.005**
Sham vs SAH+Vehicle 2	8.8667	**0.015**
Sham vs SAH+Tirofiban	0.167	1.000
SAH+Vehicle 1 vs SAH+Daltroban	10.000	0.086
SAH+Vehicle 1 vs SAH+WEB2086	1.500	1.000
SAH+Daltroban vs SAH+WEB2086	8.500	0.224
SAH+Vehicle 2 vs SAH+Tirofiban	8.833	**0.012**

Supplementary Table 25. Statistical Report of Platelet Spreading of Human SAH Platelets (Fig. 8). Univariate analysis with Dunnett's T3 post-hoc (1-2 Days Post-SAH: $F=12.513$, $p<0.001$; 4-10 Days Post-SAH: $F=64.484$, $p<0.001$).

		Mean Difference	95% Con. Interval	p-Value
1-2 Days	Saline vs Tirofiban	15.08	3.064, 27.10	**0.015**
	Saline vs A3P5PS	5.283	-0.735, 11.30	0.091
	Tirofiban vs A3P5PS	20.36	8.608, 32.12	**0.004**
4-10 Days	Saline vs Tirofiban	24.08	16.42, 31.75	**<0.001**
	Saline vs A3P5PS	0.746	-4.281, 5.773	0.970
	Tirofiban vs A3P5PS	23.34	15.13, 31.54	**<0.001**

Supplementary Table 26. Statistical Report for Platelet Counts (Supplementary Fig. 5). t-Test or Mann–Whitney U test as appropriate. All mice received injections of diphtheria toxin on days -5, -3, and -1. WT: C57BL/6J mice.

			t or U	p-Value
2-Day Study	Female	WT Sham vs WT SAH	2.043	0.059
		WT Sham vs PF4-DTR Sham	6.921	**<0.001**
		PF4-DTR Sham vs PF4-DTR SAH	1.161	0.292
		WT SAH vs PF4-DTR SAH	6.355	**<0.001**
	Male	WT Sham vs WT SAH	1.115	0.282
		WT Sham vs PF4-DTR Sham	5.721	**<0.001**
		PF4-DTR Sham vs PF4-DTR SAH	0.204	0.841
		WT SAH vs PF4-DTR SAH	5.060	**<0.001**
7-Day Study	Female	WT Sham vs WT SAH	1.944	0.057
		WT Sham vs PF4-DTR Sham	0.453	0.658
		PF4-DTR Sham vs PF4-DTR SAH	2.371	**0.031**
		WT SAH vs PF4-DTR SAH	0.878	0.386
	Male	WT Sham vs WT SAH	1.974	0.058
		WT Sham vs PF4-DTR Sham	4.719	**<0.001**
		PF4-DTR Sham vs PF4-DTR SAH	1.564	0.131
		WT SAH vs PF4-DTR SAH	0.661	0.512

References

1. Vergouwen MD, Vermeulen M, van Gijn J, Rinkel GJ, Wijdicks EF, Muizelaar JP, Mendelow AD, Juvela S, Yonas H, Terbrugge KG, Macdonald RL, Diringer MN, Broderick JP, Dreier JP and Roos YB. Definition of delayed cerebral ischemia after aneurysmal subarachnoid hemorrhage as an outcome event in clinical trials and observational studies: proposal of a multidisciplinary research group. *Stroke*. 2010;41:2391-5.
2. Dienel A, Ammassam Veettil R, Hong SH, Matsumura K, Kumar TP, Yan Y, Blackburn SL, Ballester LY, Marrelli SP, McCullough LD and McBride DW. Microthrombi Correlates With Infarction and Delayed Neurological Deficits After Subarachnoid Hemorrhage in Mice. *Stroke*. 2020;51:2249-2254.
